# Analysis and functional relevance of the chaperone TRAP-1 interactome in the metabolic regulation and mitochondrial integrity of cancer cells

**DOI:** 10.1038/s41598-023-34728-1

**Published:** 2023-05-10

**Authors:** Shrikant Purushottam Dharaskar, Khanderao Paithankar, Sreedhar Amere Subbarao

**Affiliations:** 1 CSIR-Centre for Cellular and Molecular Biology, Uppal Road, Hyderabad, Telangana 500007 India; 2 AcSIR - Academy of Scientific and Innovative Research, Ghaziabad, Uttar Pradesh India

**Keywords:** Biochemistry, Biological techniques, Cancer, Cell biology, Molecular biology

## Abstract

The 90 kDa heat shock protein, Hsp90, functions as a cancer chaperone contributing to tumor proliferation. We have encountered the mitochondrial homolog of Hsp90, the TRAP-1, regulating mitochondrial dynamics, metabolism, and tumor metastasis. Although Hsp90 is associated with a broad network of proteins regulating various cellular processes, TRAP-1-mediated cellular networks are unclear. Therefore, using TRAP-1 knockdown (KD) and overexpression (OE) systems, we compared their quantitative transcriptome (RNA Sequencing) and proteomic (LC–MS/MS) patterns to obtain molecular signatures that are altered in response to TRAP-1 KD or OE. We report TRAP-1 modulating vital metabolic pathways such as the tricarboxylic acid cycle, oxidative phosphorylation, electron transport chain, glycolysis, and gluconeogenesis. In addition, TRAP-1 facilitated the pentose phosphate pathway to shunt carbons back to glycolysis or gluconeogenesis, a much-solicited tumor response. Subsequently, we examined the TRAP-1 interactome using the tandem affinity purification system and identified 255 unique proteins. These diverse proteins appear to regulate several cellular processes, including energy metabolism, suggesting that TRAP-1, in addition to metabolic rewiring, maintains mitochondrial integrity. Our study exposes the unknown functions of TRAP-1 in cancer cells. Systematic evaluation of TRAP-1 interactors may uncover novel regulatory mechanisms in disease aggression. Since metabolic inhibitors are emerging as potential anticancer agents, our study gains importance.

## Introduction

Cellular energy metabolism grossly refers to the orchestrated oxidation of glucose through glycolysis, the TCA cycle, and oxidative phosphorylation (OXPHOS) to produce ATP linked to NADH throughput^1^,^2^,^3^. Alterations in these orchestrated processes can result in diseases like cancer^4–8^. Cancer is a polygenic disease associated with altered metabolic pathways to meet the cellular energy demand^9–11^. Cancer emergence is an adaptive cellular response to the microenvironment and the host immune system^12, 13^. Although no universal patterns of cancer are known, there is a significant overlap in mechanisms triggering the neoplastic transformation, tumor adaptation, and aggression; all of them are energy-dependent processes^14, 15^. Unlike signal transduction mechanisms that overlap considerably, the metabolic signatures are unclear^16^,^17^. Further, the trigger for altered metabolism in tumor cells is not clearly understood^18, 19^.

Normal cells use fatty acids, proteins, and glucose as primary fuel sources to produce ATP and synthesize proteins, DNA, and lipids as building blocks using conventional pathways^2^. However, tumor cells alter these pathways to meet the energy demand by rewiring metabolism through the pentose phosphate pathway (PPP) and gluconeogenesis^20, 21^. Therefore, metabolic rewiring grossly emerges in response to cellular adaptation to nutrient and oxygen deprivation to facilitate enhanced proliferative potential. Heat shock proteins (Hsps) have been identified as molecular chaperones contributing to cellular adaptations and maintaining cellular protein homeostasis^5, 22^. Among them, the high molecular weight heat shock protein, Hsp90, functions in tumor cell proliferation and metastasis^23–25^. Among Hsp90 homologs, the mitochondrial chaperone TRAP-1 regulates cellular energy metabolism, tumor metastasis, and many more^26–35^. However, TRAP-1 interacting proteins are not clearly understood but presumed that TRAP-1 acts as a tumor promoter by a few unknown or yet-to-be-identified cellular mechanisms. A few experimental studies from our laboratory and others also indicated its potential to trigger altered cellular energy metabolism to meet the energy demand of cancer cells.

Earlier, we demonstrated that TRAP-1 overexpression (OE) promotes ATP production, however, decreases mitochondrial respiration^31^. This study examined cellular networks influenced by TRAP-1 through quantitative RNA sequencing and proteomics. Subsequently, we have discussed potential interactors of TRAP-1 using a tandem affinity purification system (TAP) and their association with these identified networks. This study exposed altered metabolic networks and identified potential TRAP-1 interactors, which may contribute to altered cellular energy metabolism. Based on this, we propose TRAP-1-mediated metabolic rewiring in cancer. Since metabolic inhibitors are emerging as potential drug candidates against cancer and other diseases, our findings gain importance.

## Materials and methods

### Cell cultures and maintenance

Human neuroblastoma cells, IMR-32 (#CCL127; ATCC) after cell line authentication, were grown in Dulbecco's modified eagle medium (DMEM, #12,491-023) with 10% fetal bovine serum (FBS, #12,483-020) from Thermofisher Scientific at 37 °C in a CO_2_ incubator. The TRAP-1 overexpression (OE) and knockdown (KD) cells developed earlier were used in this study^31^. Cells grown in 25 mm culture flasks (NUNC) at a confluence of 60% were processed for RNA sequencing and proteomics analyses.

### The whole cell transcriptome library preparation and RNA sequencing analysis

#### Library preparation

The whole-transcriptome sequencing analysis provides information on coding and non-coding RNA sequences and their abundance in a given sample and enables understanding the molecular complexity^36^. The total cellular RNA isolated in triplicates from parental, OE, and KD cells was used for cDNA library preparation using TruSeq RNA Library Prep Kit v2 (Illumina) and subjected to RNA sequencing using Illumina NovaSeq6000. We used NovaSeq 6000 S4 Reagent Kit v1.5 (300 cycles) for sequencing, and Nova-Seq workflow was used to load the samples. The paired-end sequencing length was 150 bases with a coverage of 40 million reads.

#### Transcriptome analysis

Illumina adapters and low-quality reads were removed from raw sequencing reads using cutadapt. Reads with quality scores less than 20 and smaller than 36 bp were discarded. The processed reads were then mapped to the human genome GRCh38 using hisat2 with default parameters. The reads were counted using the featureCounts of Subread package. Genes with a total read count < 10 across all the samples were removed. Genes with *p* < 0.05 and log2 Fold change of > 0.5 were considered differentially expressed and used for analyses.

### Liquid chromatography (LC) with mass spectrometry (MS) analysis

The whole-cell quantitative proteomics analysis was performed for all three phenotypes, parental, KD, and OE^37^. Total cellular proteins from 1 × 10^6^ cells were collected in HEPES lysis buffer (pH 7.4), and 100 μg protein was mixed with Laemmli buffer, boiled for 5 min, and loaded onto the SDS–polyacrylamide gel electrophoresis (SDS-PAGE). After staining the proteins with Coomassie Brilliant Blue, gel slices were cut into pieces and destained using 100 mM ammonium bicarbonate and 50% acetonitrile (ACN), dehydrated using 100% ACN, and dried under vacuum. The gel slices were digested with 50 µL Trypsin Gold (#V5280, Promega, USA) prepared in 40 mM ammonium carbonate and 10% ACN for 20 h at 37 °C. After protein digestion, peptides were extracted in 100 µL 0.1% trifluoroacetic acid (TFA) in 50% ACN solution at room temperature for 1 h. The extracted peptides were purified using Zip Tips (#ZTC18M960, Millipore Biosciences, USA) with 0.1% TFA and 50% acetonitrile. The solubilized peptides were first injected into Thermo Scientific Easy-nLC 1200 equipment. The peptides were separated on a PepMap RSLC C18 column (3 µm, 100 Å, 75 µm × 15 cm, Thermofisher Scientific). Then the peptides were analyzed on Q Exactive™ Plus Hybrid Quadrupole-Orbitrap™ Mass Spectrometer (Thermofisher Scientific, USA). A scan range of 400 to 1750 m/z was applied.

### Construction of C-TAP and N-TAP expression systems

The full-length TRAP-1 cDNA (NM_016292.3) was retrieved from the online resources of the National Centre for Biotechnology Information (www.ncbi/nlm/nih.gov). A 2115 bp cDNA was PCR-amplified from the human neuroblastoma cDNA library and cloned into pNTAP B (# 240103, InterPlay N-terminal Mammalian TAP System) pCTAP A (#240104, InterPlay C-terminal Mammalian TAP System plasmid vector) from Agilent Technologies within the BamHI/EcoRI restriction sites. The recombinant expression systems were transfected into human neuroblastoma cells using the Lipofectamine™ 3000 transfection reagent (#L3000015, Thermo Fischer Scientific). The stable cells were selected with G418 (900 µg/mL) treatment for 21 days. The cells were expanded in DMEM media with 10% FBS in the presence of penicillin (100 U/mL), kanamycin (50 µg/mL), and streptomycin (50 µg/mL) at 37 °C in a humidified incubator with 5% CO_2_ supply. The cells (70 × 10^6^) were washed with PBS, trypsinized, and resuspended in isotonic buffer A (20 mM mannitol, 7 mM sucrose, 1 mM EGTA, 10 mM HEPES, pH 7.5), supplemented with 1 × protease inhibitor cocktail (#ProteCEASE, G-Biosciences). The cells were homogenized using a Dounce glass homogenizer (50 strokes). The cell lysate was centrifuged at 3500 rpm for 10 min, and the supernatant was further centrifuged at 12,000 rpm for 10 min at 4 °C (Sorval 5B, SS34 rotor). The mitochondria in the pellet fraction were lysed and used for MS/MS analysis.

### Purification of TRAP-1 interacting proteome

The mitochondrial pellet obtained in the previous section was resuspended in 10 mL of lysis buffer (Tris buffer saline pH 7.2 containing 25 mM Tris, 0.15 M NaCl, 2% CHAPS, 1 × protease inhibitor cocktail, and 1 mM PMSF), incubated on ice for 10 min. The lysed mitochondria were pelleted at 12000×*g* for 15 min at 4 °C, and the supernatant containing the mitochondrial proteins was collected in a fresh tube. To the 10 mL of mitochondrial lysate, 40 µL 0.5 M EDTA and 7 µL 14.4 M β-mercaptoethanol were added and used for affinity purification. To the 10 mL of lysate, 250 μL streptavidin resin slurry in streptavidin binding buffer (SBB) was added and incubated for 2 h at 4 °C. The mixture was centrifuged (1500×*g*, 5 min) to remove unbound proteins, and the pellet was washed in 1 mL SBB. The resin was incubated with 1 mL of streptavidin elution buffer (SEB) for 30 min at 4 °C to elute the protein complexes bound to TRAP-1. The eluent was centrifuged (1500×*g*, 5 min), and the supernatant was transferred to a fresh tube. To a 20 µL streptavidin supernatant supplement, 4 mL calmodulin binding buffer (CBB) and 125 μL calmodulin resin slurry was added to the eluent and incubated for 2 h at 4 °C followed by centrifugation at 1500×*g* for 5 min. The resin was washed by resuspending in 1 mL of CBB and centrifuged at 1500×*g* for 5 min. The resin was added with Laemmli buffer, boiled, and loaded onto gradient SDS-PAGE to separate TRAP-1 interacting proteins and for LC–MS/MS analysis.

### LC–MS/MS analysis of TRAP-1-associated proteins

After the SDS-PAGE, the proteins were stained with coomassie brilliant blue (R-250), and the bands were excised in 1 mm square pieces and transferred to 1.5 mL Eppendorf tubes. The gel pieces were allowed to shrink in 800 µL acetonitrile (15 min, × 2 times at RT) and washed with 50 mM ammonium bicarbonate solution. Excess acetonitrile was removed, and the gel pieces were dried in a vacuum for 5 min. The gel pieces were treated with 200 µL of 10 mM DTT in 50 mM ammonium bicarbonate solution and incubated for 45 min at 56 °C. The samples were cooled to room temperature, and 200 µL of freshly prepared 55 mM iodoacetamide in 50 mM ammonium bicarbonate solution was added and incubated for another 30 min at room temperature and kept in the dark. The gel pieces were then rehydrated in 200 µL of trypsin (15 ng/μL) in a solution containing 25 mM ammonium bicarbonate and 1 mM calcium chloride and incubated at 37 °C for 16 h.

The peptides from the gel pieces were trypsin digested at 37 °C overnight and subsequently washed on a vibrator shaker for 1 h with 650 µL of 5% formic acid in 30% acetonitrile and sonicated for 5 min. The peptides were extracted in 5% formic acid containing 30% acetonitrile, transferred to a fresh 1.5 mL Eppendorf tube, and the gel pieces were re-extracted with 150 µL of 5% formic acid in 30% acetonitrile. The extracted peptides were centrifuged at 16,000 rpm for 40 min, and the supernatant was collected into a fresh Eppendorf tube. The peptides were then vacuum-dried and stored at − 30 °C for further use. The peptides were reconstituted in 15 μL of 5% acetonitrile containing 0.1% TFA, desalted with a C18 zip-tip. The peptides slowly spurged through the tip to bind to the resin. The peptides bound to the C18 tip were then washed with 0.1% TFA, followed by 5% acetonitrile and 0.1% TFA in distilled water. Finally, the peptides were eluted with 25 µL of 50% acetonitrile. Subsequently, the desalted peptides were vacuum dried, stored at − 30 °C, or used for LC–MS/MS analysis. The spectra obtained were converted to protein identities and used for STRING and ShinyGo 0.76.2 analyses.

### Analysis of transcriptome and proteome data

For the functional enrichment analysis, the clusterProfiler was used for GO term enrichment, followed by the Biological process analysis using the KEGG pathway database. The data from NGS and proteome were sorted based on fold change in gene/protein expression. We represented the RNASeq and the proteomic data on a log scale using log2 fold change, considering a *p*-value less than 0.05 as significant. Since this representation is logarithmic and not linear, control values are not normalized to 1.0. Therefore, a value more than 0.0 is considered for increased expression, and a value less than 0.0 is considered for decreased expression. We used Thermo Proteome discoverer software (2.2.0.388) for the label-free quantification of the protein IDs. The contaminants, such as keratin, were removed from the raw data (in the case of the proteome), and the resulting data were normalized against parental cells. The upregulated and downregulated transcripts/proteins from OE and KD were used for analysis using STRING (www:http//string-db.org) and ShinyGo 0.76.2 (www:http//bioinformatics.sdstate.edu). Similarly, we analyzed the proteomics data obtained from the TAP system too. The data also generated from KEGG for different pathways^38–40^.

### Evaluation of NGS and LC–MS/MS data

The NGS and LC–MS/MS data were analyzed by quantitative polymerase chain reaction (qPCR) using appropriate primers (Table S1). For PCR experiments, total RNA was isolated from parental, KD, and OE cells using the Trizol reagent (#15596-018, Invitrogen). From 1 μg RNA of each, a cDNA library was prepared using PrimeScript™ 1st strand cDNA Synthesis Kit (#6110A; Takara). In the case of quantitative PCR, TB Green® Premix Ex Taq™ II (#RR82LR, Takara) was used to amplify the cDNA as per the manufacturer protocol. Equal volumes of cDNA library from parental, KD, and OE cells were used to quantify genes of interest using a quantitative real-time PCR machine (ViiA 7, Applied Biosystems). For immunoblot analyses, total cell lysates from parental, KD, and OE cells were prepared using RIPA lysis buffer (pH 7.4). Total protein was estimated using the BCA method, and 50 μg of cell lysate was added with Laemmli buffer, denatured at 50 °C for 5 min, and loaded on 10% SDS-PAGE. Samples were not boiled since the OXPHOS intermediates are sensitive to denaturation at high temperatures. The proteins are transferred to the nitrocellulose membrane and used for immunoblotting with appropriate antibodies.

## Results

### RNA sequencing analysis of TRAP-1 KD and OE cells

**Figure 1:**
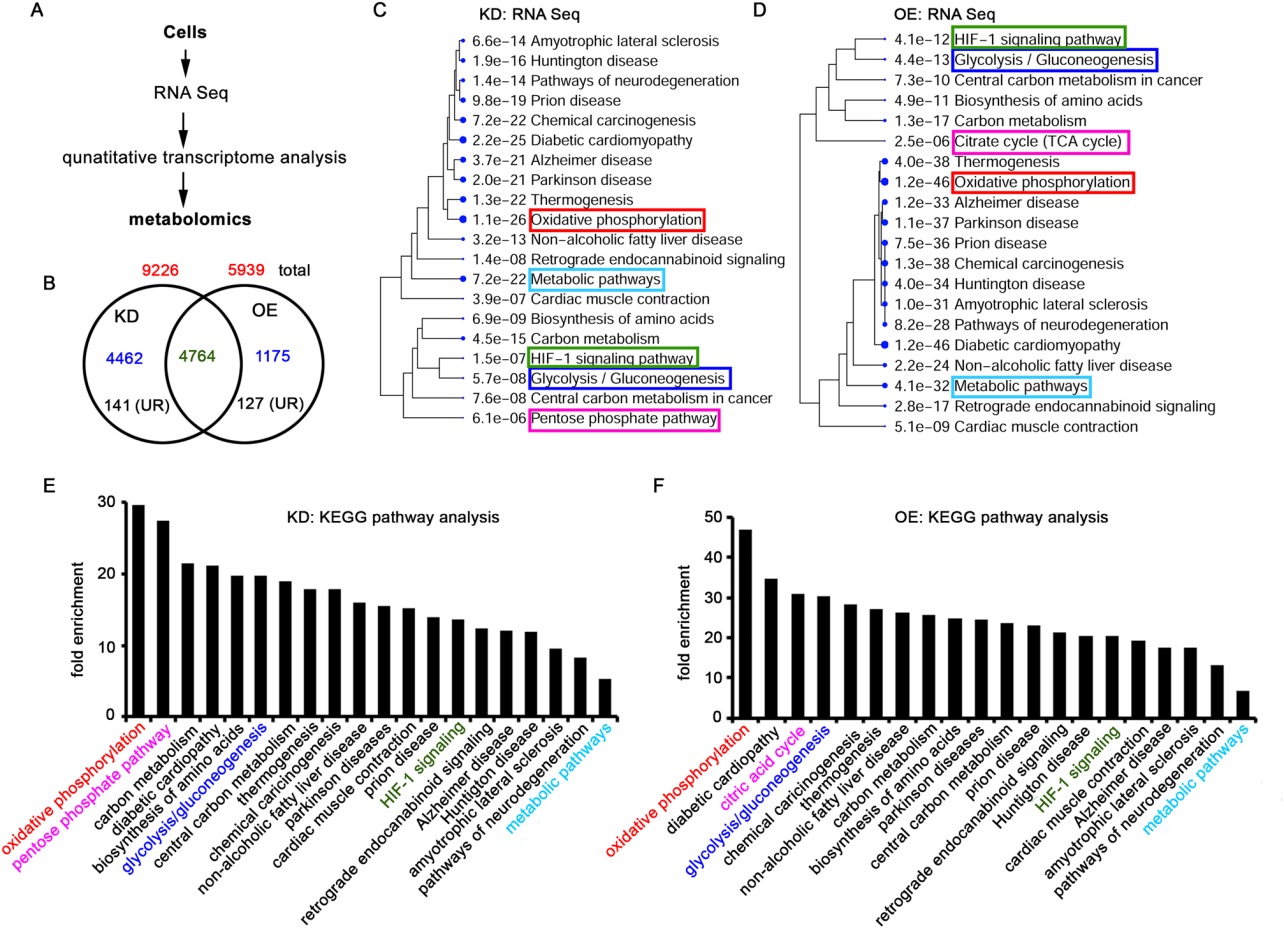
The transcriptome analysis (RNA sequencing) of OE and KD cells. (**A**) The schematic representation of quantitative RNA sequencing followed by metabolome analysis. The total RNA from parental, KD, and OE cells was isolated, the mRNA library was generated using TruSeq RNA Library Prep Kit v2 (Illumina), and subjected to RNA sequencing using Illumina NovaSeq6000. The transcript IDs obtained from KD and OE were normalized with parental cells, and the upregulated IDs were analyzed by ShinyGO 0.76. (**B**) The KD and OE IDs were subjected to Venn Diagram analysis, and the unique and overlapping IDs (> 0.5 folds) are represented. The numbers in red color indicate the total IDs from KD and OE. The numbers in blue indicate unique IDs in each phenotype, and the green color indicates common IDs. The numbers in black color are upregulated ones in KD and OE. UR: upregulated. Note that only upregulated IDs are used for pathway analysis. (**C**,**D**) The KEGG pathway analysis of upregulated IDs from KD (**C**) and OE (**D**) cells. The e-value represents the significance, and the number before each pathway indicates the number of hits we obtained from this study. Larger the size of the dot, the higher the significance. (**E,F**) The KEGG pathway analysis shows fold enrichment from each pathway. Higher fold enrichment suggests increased expression of genes in each pathway. Note the increased number of genes in oxidative phosphorylation, the TCA cycle, and glycolysis/gluconeogenesis in OE compared to KD cells. IDs: identification numbers.

The NGS-based RNA sequencing provides gross information on coding (e.g., biomarkers, epigenetic patterns, multidrug resistance, tumor heterogeneity, altered metabolism, and tumor antigens from the whole transcriptome) RNA transcripts. Therefore, we have performed RNA-sequencing of the parental, KD, and OE human neuroblastoma cells using NovaSeq 6000 system and analyzed the data for potential metabolic alterations associated with TRAP-1 (GEO record no. GSE229114). We have normalized the KD and OE data with parental, and the new IDs not listed in Uniprot are excluded, and the information obtained from individual phenotypes is being reported (Fig. 1A, Table S2). From the KD cells, we got 9226 IDs and out of which 4462 were found to be unique. From the OE cells, we obtained 5939 IDs, out of which 1175 were found to be unique, and 4764 were shared in KD and OE cells (Fig. 1B, Table S2).

From 4462 IDs of KD, 141 showed upregulation, and from 1175 IDs of OE, 127 were found to be upregulated. The KEGG analysis of 141 IDs from KD showed metabolic pathways, pentose phosphate pathway, glycolysis/gluconeogenesis, HIF1α signaling pathway, and oxidative phosphorylation in the increasing order of significance (Fig. 1C). Similarly, the KEGG analysis of 127 IDs using ShinyGO tool highlighted glycolysis/gluconeogenesis, metabolic pathways, the HIF1α signaling pathway, the TCA cycle, and oxidative phosphorylation in the increasing order of significance (Fig. 1D). Concerning fold enrichment, the KD cells showed oxidative phosphorylation, pentose phosphate pathway, glycolysis/gluconeogenesis, HIF-1 signaling, and metabolic pathways in decreasing order (Fig. 1E). Whereas, OE cells showed oxidative phosphorylation, citric acid cycle, glycolysis/gluconeogenesis, HIF-1 signaling, and metabolic pathway in decreasing order (Fig. 1F).

**Figure 2:**
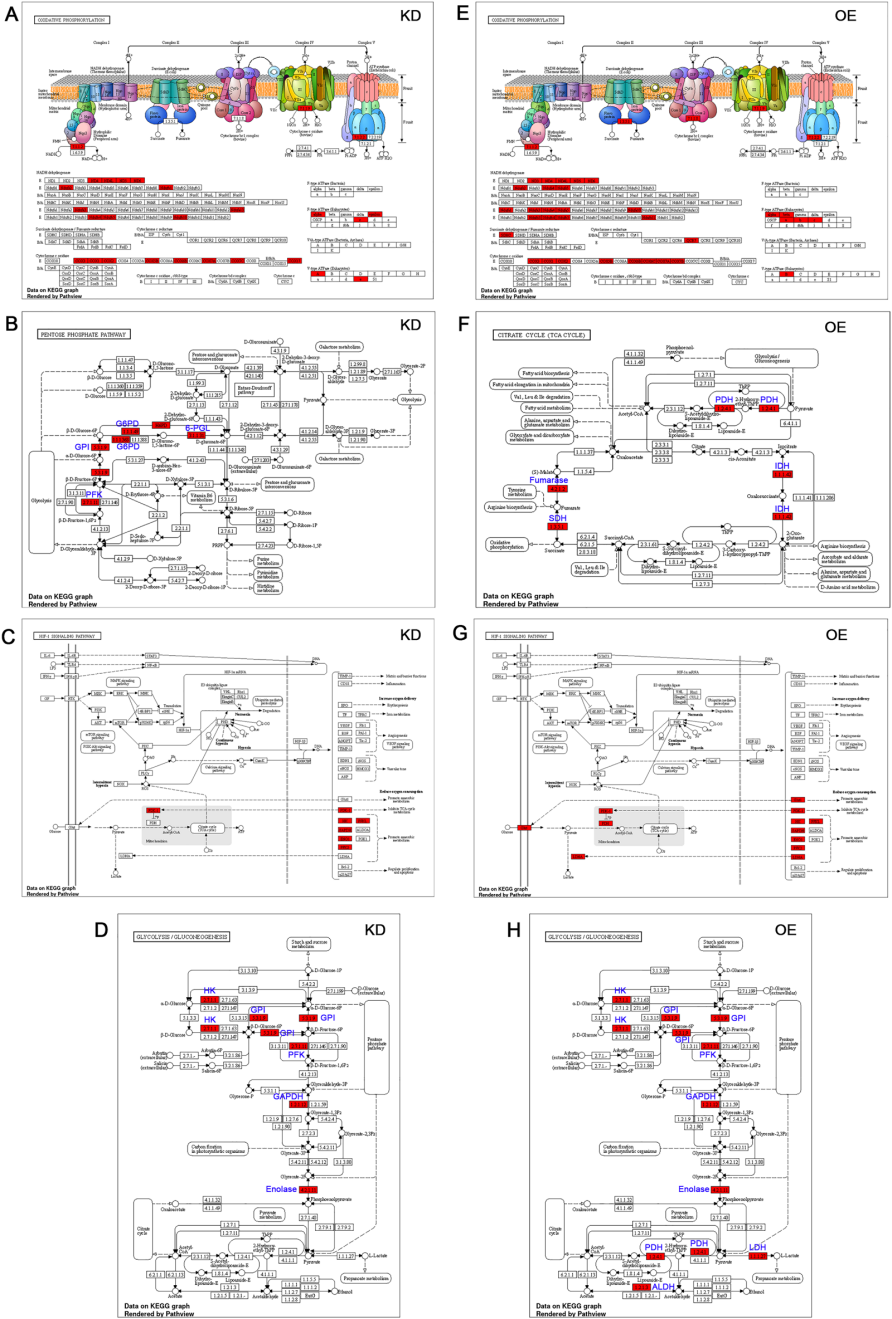
KEGG analysis of NGS data from OE and KD cells. The major pathways that showed fold enrichment was used to identify upregulated genes. (**A**,**E**) The schematic representation shows the five complexes of oxidative phosphorylation (OXPHOS). The red color highlighted were the genes that showed upregulation compared to parental cells. (**A**) represents KD, and (**E**) represents OE. Note that OE cells show an increased number of genes compared to KD. (**B**) schematic representation of pentose phosphate pathway (PPP) in KD cells. The red color EC numbers indicate genes picked from this study. The blue color text indicates the name of the enzyme in the pathway. (**C**) Schematic representation of HIF1α signaling pathway in KD cells. The red color text indicates genes picked from this study. (**D**) Schematic representation of glycolysis/gluconeogenesis pathways in KD cells. The red color EC numbers indicate genes picked from this study. The blue color text indicates the name of the enzyme in the pathway. (**F**) Schematic representation of citric acid cycle in OE cells. The red color EC numbers indicate genes picked from this study. (**G**) Schematic representation of HIF1α signaling pathway in OE cells. The red color text indicates genes picked from this study. (**H**) Schematic representation of glycolysis/gluconeogenesis pathways in OE cells. The red color EC numbers indicate genes picked from this study. The blue color text indicates the name of the enzyme in the pathway.

Further, from the maps generated using KEGG database^38–40^ we found that 27 genes from OXPHOS, seven from the pentose phosphate pathway, six from the HIF1α pathway, and five from glycolysis/gluconeogenesis were overexpressed in KD cells (Fig. 2A–D). We did not observe any TCA cycle intermediates being overexpressed in these cells. In comparison, OE cells showed 38 genes from OXPHOS, four from the TCA cycle, ten from the HIF1α pathway, and eight from glycolysis/gluconeogenesis. We did not observe any gene upregulation from the pentose phosphate pathway (Fig. 2E–H). The OE cells showed a significant increase in succinate dehydrogenase (*SDH*; 0.06 folds). The fumarase (0.08 folds), pyruvate dehydrogenase (*PDH*; 0.91 folds), and isocitrate dehydrogenase (*IDH*; 0.79 folds) from the TCA cycle suggests enhanced TCA in OE cells compared to KD cells. The increase in the TCA cycle in OE cells means increased mitochondrial functions. An increase in gene expressions of glucose-6-phosphate dehydrogenase (*G6PD*; 0.85 folds; *G6PD* variant; 0.18 folds), 6-phosphogluconolactonase (*6-PGL*; 0.19 folds), phosphofructokinase (*PFK*; 0.54 folds) in KD cell indicate active pentose phosphate pathway. An increase in *glut* and *glut1* (glucose transporters) and *LDHA* expressions suggests that OE cells can also make cellular energy from the anaerobic metabolism. An increase in pyruvate dehydrogenase (*PDH*; 0.91 folds; aldehyde dehydrogenase (*ALDH*; 0.26 folds), and lactate dehydrogenase (*LDH*; 0.28 folds) suggest the potential of OE cells to make acetyl CoA from glycolysis and fatty acid metabolism.

### Effect of TRAP-1 KD and OE on the altered cellular proteome

**Figure 3:**
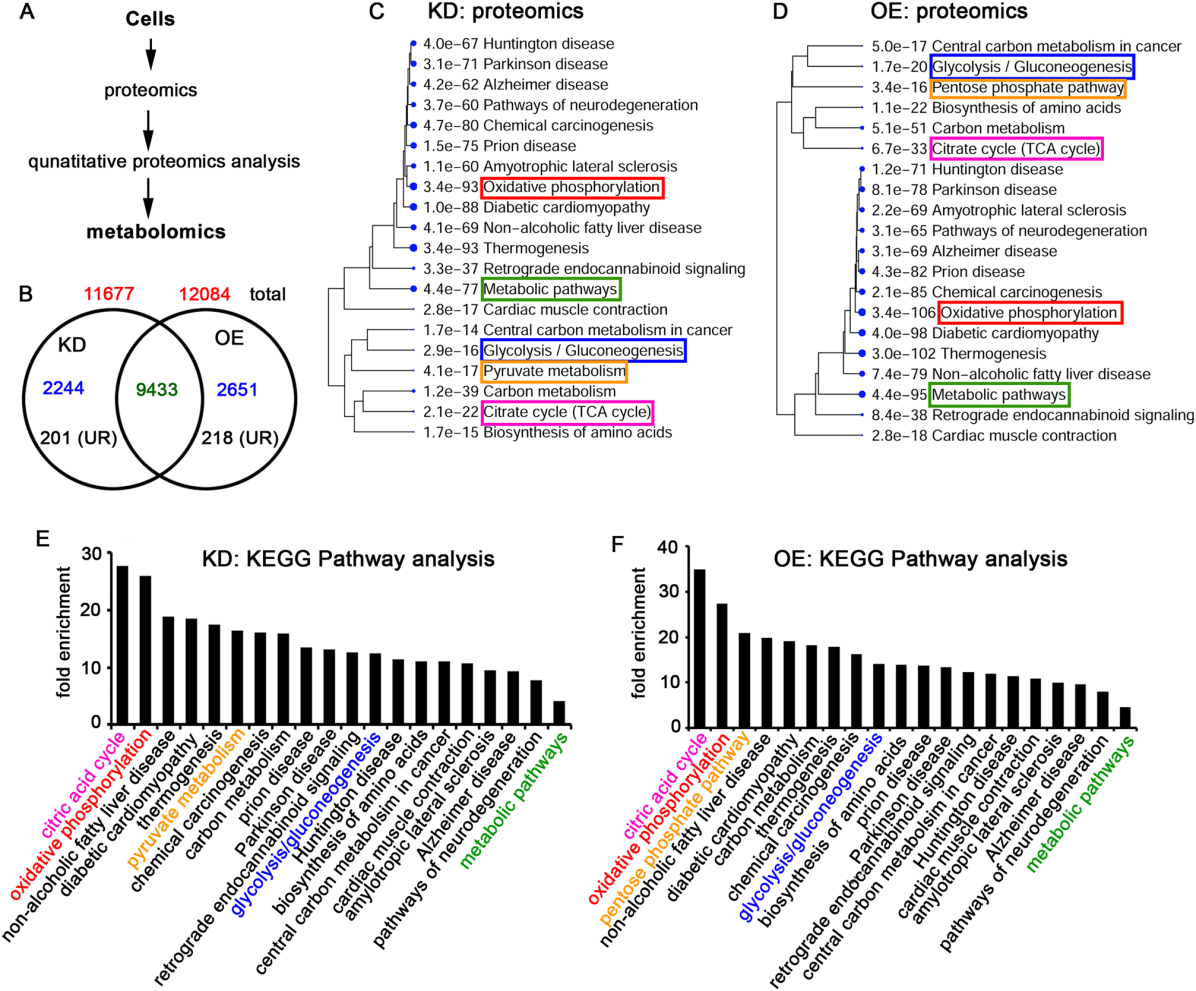
The proteomic analysis of OE and KD cells. (**A**) The schematic representation of quantitative proteomics followed by metabolome analysis. The total cell lysates from parental, KD, and OE cells were isolated and subjected to LC–MS/MS analysis using QExactive HF. The protein IDs obtained from KD and OE using Thermo Proteome Discoverer version 2.2.0.388 were normalized with parental cells, and the upregulated IDs were analyzed by ShinyGO 0.76. (**B**) The KD and OE IDs were subjected to Venn Diagram analysis, and the unique and overlapping IDs are represented. The numbers in red color are the total IDs. The numbers in blue are unique IDs in each phenotype, and the green color indicates common IDs. The numbers in black color are upregulated proteins in KD and OE. UR: upregulated. Note that upregulated IDs are used for pathway analysis. (**C**,**D**) The KEGG pathway analysis of upregulated IDs from KD (**C**) and OE (**D**) cells. The e-value represents the significance, and the number before each pathway indicates the number of hits we obtained from this study. Larger the size of the dot, the higher the significance. (**E**,**F**) The KEGG pathway analysis shows fold enrichment from each pathway. Higher fold enrichment suggests upregulated proteins in each pathway. Note the increased number of proteins in oxidative phosphorylation, the TCA cycle, pentose phosphate pathway, and glycolysis/gluconeogenesis in OE compared to KD cells. IDs: identification numbers.

The quantitative proteomics analysis provides information on the functional proteins associated with cell types. Therefore, we performed cellular proteome analysis using Q Exactive HF and analyzed the data (Fig. 3A). After normalizing the IDs with parental cells, we obtained 11,677 and 12,084 IDs for KD and OE cells, respectively. While 9433 were present in both cell types, 2244 were unique to KD cells, and 2651 were unique to OE cells (Fig. 3B, Table S3). From the 2244 unique IDs from KD cells, 201 were found to be linked to energy metabolism. Out of 2651 IDs from OE, 218 are found to be associated with energy metabolism. The KEGG analysis using ShinyGO shows KD cells are efficient with oxidative phosphorylation, metabolic pathways, citric acid cycle, pyruvate metabolism, and glycolysis/gluconeogenesis (Fig. 3C). The KEGG analysis of OE cells showed increased oxidative phosphorylation, metabolic pathways, citric acid cycle, glycolysis/gluconeogenesis, and pentose phosphate pathway (Fig. 3D). The fold enrichment analysis had highlighted the citric acid cycle, oxidative phosphorylation, pyruvate metabolism, glycolysis/gluconeogenesis, and metabolic pathways in the decreasing order in KD cells (Fig. 3E). Whereas, OE cells showed increased citric acid cycle, oxidative phosphorylation, pentose phosphate pathway on priority, followed by glycolysis/gluconeogenesis, and metabolic pathway (Fig. 3F). The metabolic profiling of protein expression in OE suggests enhanced glycolysis/gluconeogenesis, mitochondrial functions, and pentose phosphate pathway.

**Figure 4:**
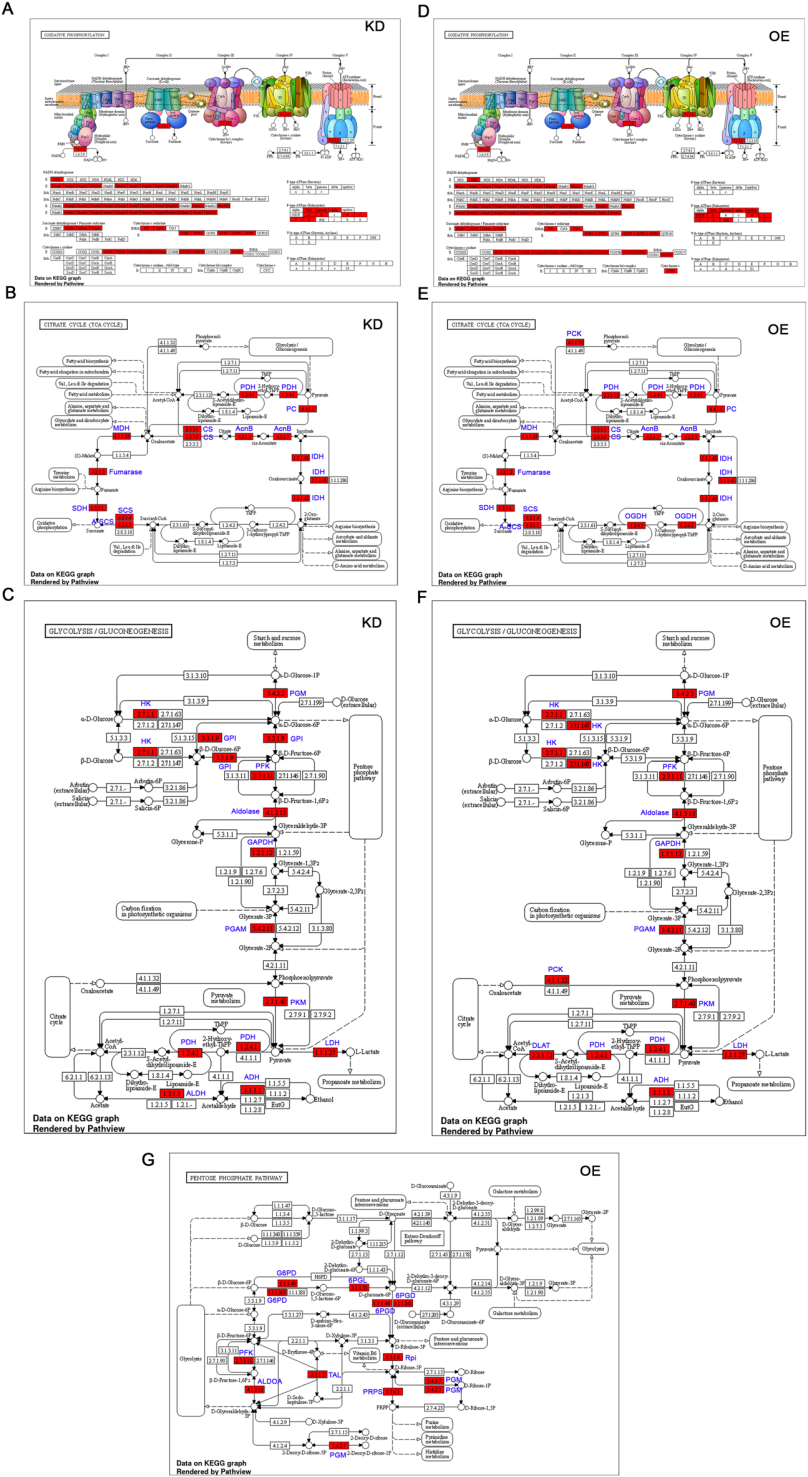
KEGG analysis of proteomics data from OE and KD cells. (**A**,**D**) Schematic representation of oxidative phosphorylation (OXPHOS) shows five complexes. The red color highlighted were the genes that showed upregulation compared to parental cells. (**A**) represents KD, and (**D**) represents OE. Note that OE cells show an increased number of proteins compared to KD. (**B**,**E**) Schematic representation of citric acid cycle in KD and OE cells. (**B**) represents KD, and (**E**) represents OE cells. The red color EC numbers indicate the EC number of the proteins picked from this study. The blue color text indicates the name of the enzyme in the pathway. (**C**,**F**) Schematic representation of glycolysis/gluconeogenesis pathways in KD and OE cells. The red color text indicates the protein IDs picked from this study. The red color EC numbers indicate genes picked from this study. The blue color text indicates the name of the enzyme in the pathway. (**C**) represents KD, and (**F**) represents OE cells. (**G**) Schematic representation of pentose phosphate pathway (PPP) in OE cells. The red color text indicates proteins picked from this study. The blue color text indicates the name of the enzyme in the pathway.

The KEGG analysis highlighted the different proteins that are upregulated in each pathway^38^,^39^,^40^. The oxidative phosphorylation highlighted 65 IDs, the citric acid cycle highlighted 10 IDs, and glycolysis/gluconeogenesis highlighted 12 IDs from KD cells (Fig. 4A–C). The OE cells highlighted 74 IDs from oxidative phosphorylation, 10 from the citric acid cycle, 12 from glycolysis/gluconeogenesis, and 8 from the pentose phosphate pathway (Fig. 4D–G). From the KEGG analysis, we observed an increase in phosphoenolpyruvate carboxykinase (PEPCK; 0.77 folds), dihydro lipoyl lysine-residue acetyltransferase, a component of pyruvate dehydrogenase (PDH; 1.31 folds), and oxoglutarate dehydrogenase, a key enzyme of metabolic flux in the TCA cycle and is closely related to pyruvate dehydrogenase complex (OGDC; 1.73 folds) in OE cells compared to KD cells (Fig. 4B,E).

In the case of glycolysis/gluconeogenesis, we observed the presence of phosphoenolpyruvate carboxykinase (PEPCK) and the absence of glucose-6-phosphate isomerase (GPI), a key enzyme of glycolysis, suggesting a potential contribution to pentose phosphate pathway (Fig. 4C,F). However, the OE cells additionally exhibited the involvement of the pentose phosphate pathway highlighting glucose-6-phosphate dehydrogenase (G6PD; 11.5 folds), fructose-bisphosphatase (6PGL; 2.51 folds), phosphogluconate dehydrogenase (6PGD; 1.9 folds), ribose-5-phosphate isomerase (Rpi; 0.6 folds), ribose-phosphate diphosphokinase (PRPS; 1.12 folds), phosphopentomutase (PGM; 1.0 folds), phosphoglucomutase (PGM variant; 2.1 folds), transaldolase (TAL; 1.6 folds), 6-phosphofructokinase (PFK; 4.4. folds), and fructose-bisphosphate aldolase (ALDOA; 2.0 folds). Unlike NGS, we did not observe an enhanced pentose phosphate pathway in KD. Therefore, the metabolic profiling of OE suggests enhanced glycolysis and mitochondrial functions followed by refueling (Fig. 4G).

### Evaluation of NGS and proteomics data

**Figure 5:**
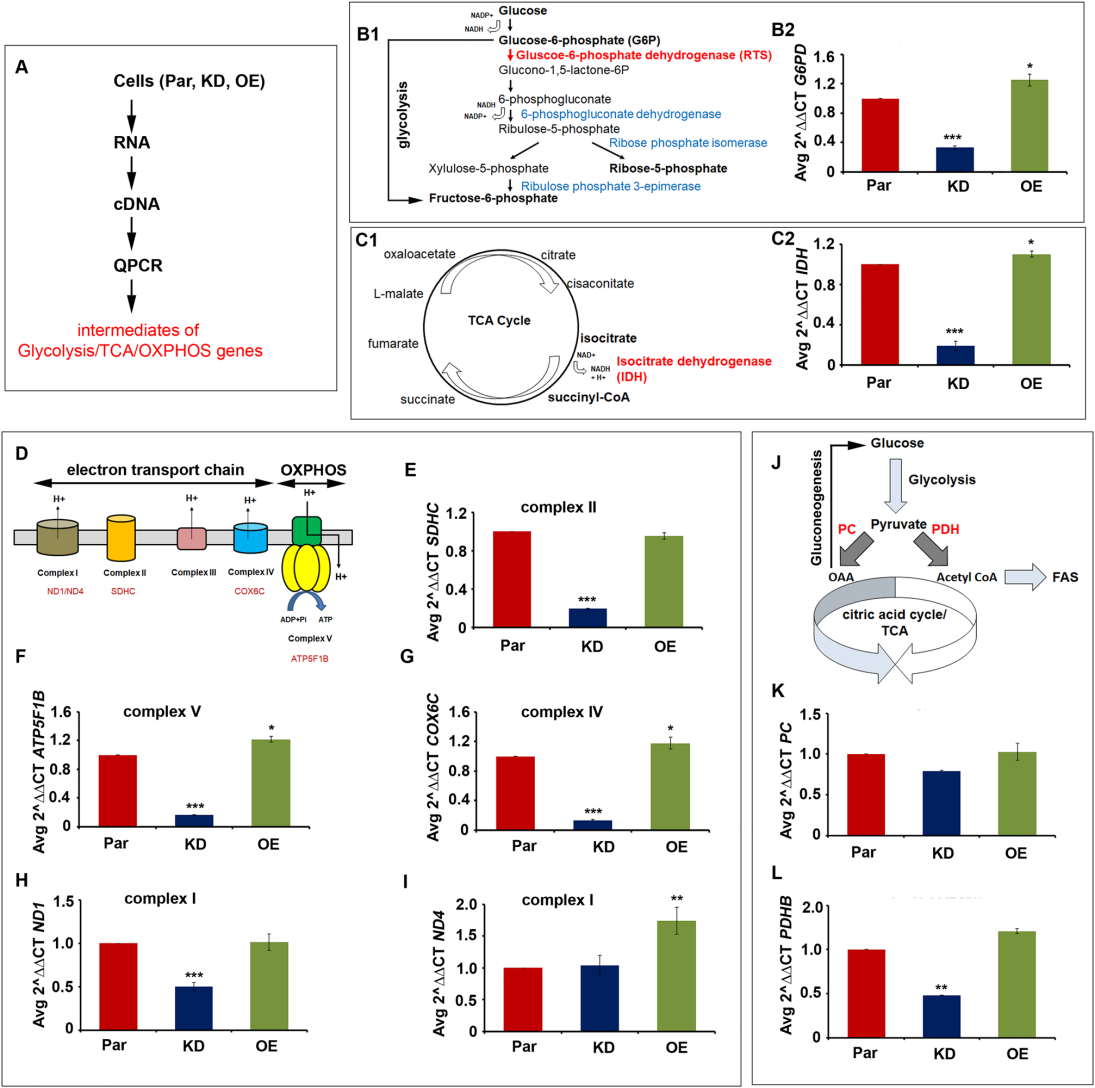
Evaluation of RNA sequencing and proteomics data by quantitative polymerase chain reaction (qPCR). (**A**) Schematic representation showing cell types used for RNA isolation, cDNA library construction, and ﻿qPCR. (**B1**) Schematic representation of pentose phosphate pathway (PPP). The red color text indicates the rate-limiting enzyme in the PPP. The blue color text indicates the enzymes involved in the execution of PPP. (**B2**) ﻿qPCR analysis of Glucose-6-phosphate dehydrogenase (*G6PD*) in parental (Par), TRAP-1 knockdown (KD), and TRAP-1 overexpression (OE) cells. (**C1**) The schematic representation of the tricarboxylic acid or citric acid cycle. The red color text indicates the key enzyme of the cycle. (**C2**) ﻿qPCR analysis of isocitrate dehydrogenase (*IDH*) in parental (Par), TRAP-1 knockdown (KD), and TRAP-1 overexpression (OE) cells. (**D**) The schematic representation of electron transport chain and oxidative phosphorylation. The red color text indicates the genes that are used for ﻿qPCR analysis. (**E**)﻿ qPCR analysis of complex II enzyme *succinate dehydrogenase C* (*SDHC*). (**F**) ﻿qPCR analysis of complex V enzyme *ATP synthase F1 subunit beta* (*ATP5F1B*). (**G**) ﻿qPCR analysis of complex IV enzyme *cytochrome C oxidase subunit 6C* (*COX6C*). ﻿qPCR analyses of complex I enzymes *NADH-ubiquinone oxidoreductase chain-1* (*ND1*) (**H**) and *NADH-ubiquinone oxidoreductase chain-4* (*ND4*) (**I**). (**J**) The schematic representation shows how pyruvate carboxylase (PC) and pyruvate dehydrogenase (PDH) enzymes link glycolysis, the citric acid cycle, gluconeogenesis, and fatty acid synthesis (FAS). ﻿qPCR analyses of *PC* (**K**) and *PDHB* (**L**) enzymes in three phenotypes. ***indicates *P* < 0.001; **indicates *P* < 0.01; * indicates *P* < 0.05.

The RNA sequencing and proteomics data suggested increased mitochondrial functions in OE cells compared to KD cells. However, we observed decreased oxygen consumption rate in OE cells^31^. Therefore, we like to examine the mechanisms that fuel mitochondria in these cells. We chose critical molecules highlighted in our RNA sequencing from four metabolic pathways to explore the metabolic differences between KD and OE cells for expression analysis (Fig. 5A). From the glycolysis, we chose *G6PD* as the rate-limiting step. We found that in KD cells, its expression is decreased by 0.67 folds, and OE cells showed a 0.25 fold increase (Fig. 5B1,B2). From the citric acid cycle/TCA, we chose *IDH* as the rate-limiting step. We found 0.81 folds decrease in KD cells compared to 0.1 folds increase in OE cells (Fig. 5C1,C2). From complex II (OXPHOS), we chose *SDHC*. It may be noted that SDH is a dual enzyme that works both in TCA and OXPHOS (Fig. 5D). We found 0.81 folds decrease in KD cells. Interstingly, there is no difference in *SDHC* expression in parental and TRAP-1 OE cells (Fig. 5E). From complex V (the electron transport chain), we chose *ATP5F1B* and found 0.84 folds decrease in KD cells compared to 0.22 fold increase in OE cells (Fig. 5F). From complex IV, we chose *COX6C*. We observed 0.86 folds decrease in KD cells and a 0.18-fold increase in OE cells (Fig. 5G). Then, we examined the integrity of complex I mitochondrial genes *ND1* and *ND4* and found that OE cells show enhanced expression compared to KD cells (Fig. 5H,I).

Since we also observed increased glycolysis/gluconeogenesis in OE cells, we speculated that the pyruvate produced from glycolysis might fuel the citric acid cycle in these cells. Pyruvate can result in two cell fates, (1) refueling glycolysis by activating gluconeogenesis through pyruvate carboxylase (PC) or fueling the citric acid cycle through pyruvate dehydrogenase (PDH) or (2) PDH, in addition to refueling the citric acid cycle, it can also contribute to fatty acid synthesis (FAS)^41^, Fig. 5J). Therefore, we have examined the expression levels of PC and PDH in parental, KD, and OE cells. There was a significant decrease in PC (0.21 folds) and PDH (0.52 folds) in KD cells, whereas we observed an increase in PC (0.03 folds) and PDH (0.21 folds) in OE cells (Fig. 5K,L). These results suggest that OE cells may exhibit an increased citric acid cycle or FAS to fuel cells. Therefore, subsequent studies may be required to confirm this.

### Identification of TRAP-1 interacting proteome exposes the significance of TRAP-1 in regulating mitochondrial energy metabolism

**Figure 6:**
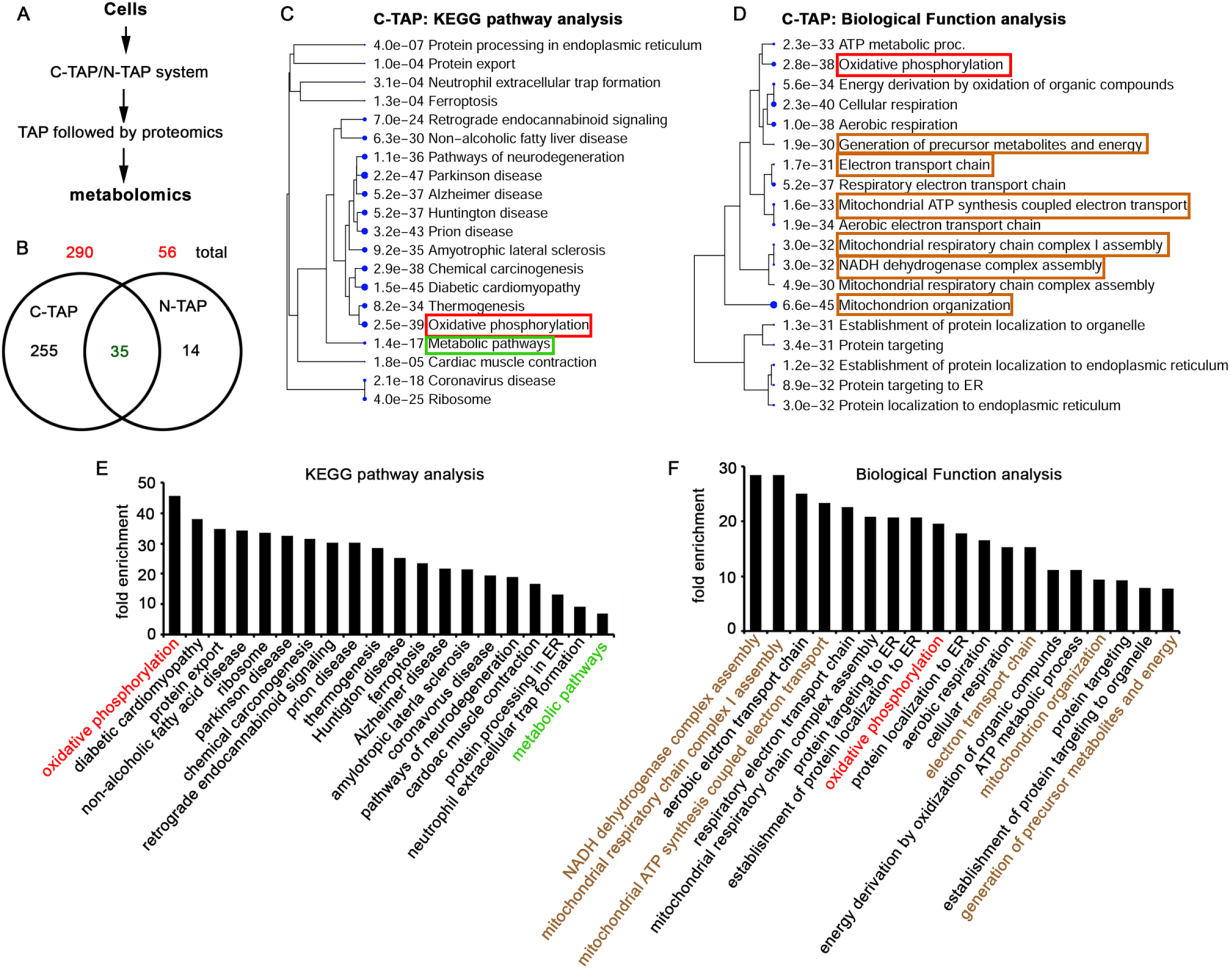
The C-TAP and N-TAP proteomics analysis from parental cells. (**A**) The schematic representation of proteomics analysis of C-TAP (mitochondrial) and N-TAP (extramitochondrial) system. Cell lysates after C-TAP or N-TAP stable transfections were subjected to proteomics analysis using Q Exactive HF mass spectrometer. The IDs obtained from each system using Thermo Proteome Discoverer version 2.2.0.388 were analyzed by ShinyGO 0.76. (**B**) The C-TAP and N-TAP IDs were subjected to Venn Diagram analysis, and the unique and overlapping IDs are represented. The numbers in red color indicate the total IDs from C-TAP and N-TAP. The numbers in black color indicate unique IDs in each phenotype and are used for pathway analysis. The common IDs are indicated by green color. (**C**) The KEGG pathway analysis of C-TAP IDs. (**D**) The biological function analysis of C-TAP IDs. The e-value represents the significance, and the number before each pathway indicates the number of hits we obtained from this study. Larger the size of the dot, the higher the significance. (**E**) The KEGG pathway analysis shows fold enrichment from each pathway. (**F**) The biological function analysis shows fold enrichment from each process. Higher fold enrichment suggests an increased number of proteins in each pathway.

RNA sequencing and quantitative proteomics results suggested that TRAP-1 OE cells exhibit enhanced mitochondrial functions and, more specifically, improved TCA cycle functions. The enhanced TCA can have two outcomes: increasing precursor metabolites or contributing to cellular energy metabolism to generate ATP through ETC and OXPHOS^42^. Having observed the effect of TRAP-1 OE on the metabolic regulatory mechanisms, we want to determine how TRAP-1 achieves this. Unlike the Hsp90 chaperone, the TRAP-1 interactome is unavailable; hence, its interacting partners are unknown. Therefore, in the present study, we have adopted a tandem affinity purification (TAP) system that allows the rapid purification of protein complexes under native conditions^43^. As explained in materials and methods, we have constructed two TAP systems, C-TAP and N-TAP, where the C-TAP majorly pulls down mitochondrial proteins. In contrast, it is presumed that N-TAP can pulldown extramitochondrial proteins. The human neuroblastoma cells were stably transfected with these systems, and the cell lysates were subjected to affinity purification followed by quantitative proteomic analysis using Q Exactive HF mass spectrometer (Fig. 6A, Fig. S1A).

The proteomics analyses of affinity purified fractions containing the protein complexes interacting with TRAP-1. While C-TAP pulls down the complexes within mitochondria, N-TAP pulls down the complexes from extramitochondrial sources. We obtained 290 IDs with C-TAP from the quantitative proteomics analyses and 56 with N-TAP (Table S4). Thirty-five IDs were present in both C-TAP and N-TAP systems. We separated 255 unique IDs from the C-TAP system and 14 from the N-TAP system (Fig. 6B). The KEGG analysis of 255 IDs from C-TAP shows most of the pathways related to mitochondrial health and metabolism (Fig. 6C). In comparison, 14 IDs from N-TAP highlighted the pathways related to oxidative stress and antioxidant systems and are nonspecific to mitochondrial metabolism (Fig. S1B). The biological function analysis of C-TAP cells showed increased oxidative phosphorylation along with the electron transport (Fig. 6D). The fold enrichment analysis highlighted oxidative phosphorylation from KEGG analysis (Fig. 6E). In contrast, the fold enrichment analysis from biological function revealed the electron transport chain complexes in the higher order (Fig. 6F). Interestingly, these results are in agreement with our NGS and proteomics evaluation results that *ND1* and *ND4* expressions have increased in OE cells (Fig. 5H,I).

**Figure 7:**
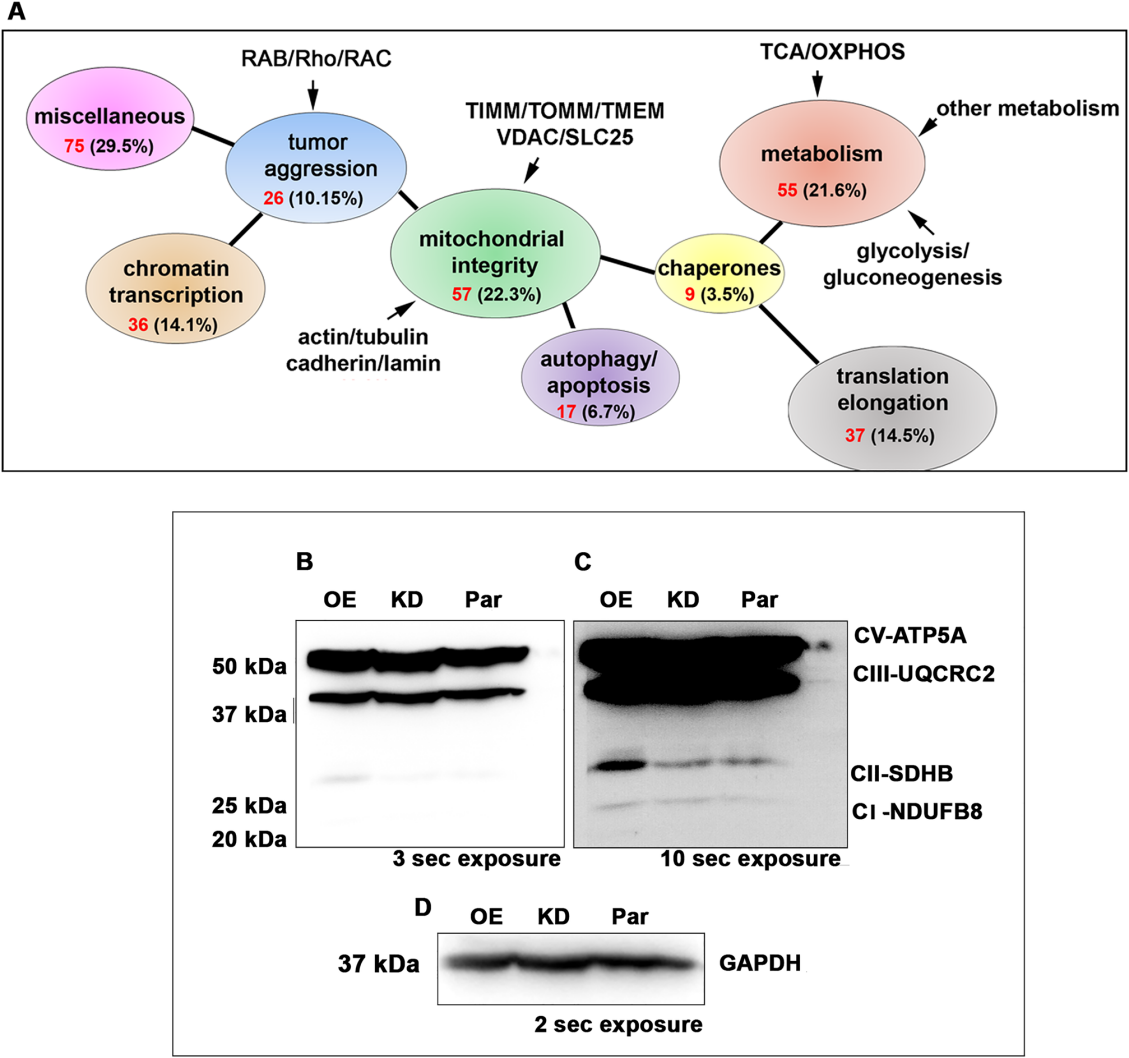
Evaluation of proteomics/C-TAP data. (**A**) Functional assignment of 255 IDs from the C-TAP system into various cellular processes. Note that 50% of the IDs cover mitochondrial integrity and metabolism, while the rest span transcriptional and translational regulation, cell death, and other functions. The percentage in each circle represents the share of that particular function, and the red number in each circle represents the number of proteins from that particular pathway. (**B**) Immunoblot analysis of parental, TRAP-1 knockdown (KD), TRAP-1 overexpression (OE) whole cell lysates with electron transport chain (ETC), and oxidative phosphorylation (OXPHOS)-specific proteins on shorter exposure. (**C**) Immunoblot analysis of ETC and OXPHOS proteins on longer exposure.** (D**) Immunoblot analysis of housekeeping gene on shorter exposure. GAPDH. Par: parental cells; KD: TRAP-1 knockdown cells; OE: TRAP-1 overexpression cells; sec: seconds.

Using the STRING analysis, we have annotated 255 IDs obtained from the C-TAP and segregated them based on functions. These are tumor promoters (26 IDs), constituting 10.15%; mitochondrial integrity-associated proteins (57 IDs), constituting 22.3%; mitochondrial metabolism-associated proteins (55 IDs), constituting 21.6%; proteins associated with DNA binding and chromatin transcription (36 IDs), constituting 14.1%; protein translation and elongation (37 ID), constituting 14.5%; cell death regulators (17 IDs), constituting 6.7%; chaperones and cochaperones (9 IDs), constituting 3.5%; miscellaneous (75 IDs), constituting 29.5%. Within the metabolism-associated ones, 76.4% are from the OXPHOS, 14.6% are from glycolysis/gluconeogenesis, and 5.5% are from other metabolic pathways. Of the mitochondrial-integrity-associated ones, 29.8% are the membrane receptors, transport, and pore transition proteins; 19.3% are from cytoskeletal proteins such as actin, tubulin, cadherin, and lamin (Table 1, Fig. 7A). The STRING analysis using TRAP-1 as bait highlighted its potential involvement in oxidative phosphorylation and the TCA cycle. Among the top 20, PINK1 and SDH have been studied to some extent. Using the public database, we have mapped TRAP-1 interactors (Table S5, Fig. S2)^44, 45^. Therefore, the rest of them are either predicted or hypothesized. The C-TAP system has highlighted several of them, including the ones already identified, strengthening that our TAP system could pick up the interactions in their native state.

**Table 1:** TRAP-1 interacting protein IDs identified by C-TAP system.

ID	Name	Function
Tumor promoters		
DDONT	Dolichyl-diphosphooligosaccharide–protein glycosyltransferase 48 kDa subunit	Tumor migration and metastasis
FAM162A	Family with sequence similarity 162 member a	PTP, mitochondrial integrity
GNA12	Guanine nucleotide-binding protein G(i) subunit alpha-2	Tumor progression
GNAS	Guanine nucleotide-binding protein G(s) subunit alpha isoforms XLas	Tumor progression
HRNR	Hornein	Tumor promoter (S100 family member)
JUP	Junction plakoglobin	Tumor metastasis
MAGEA8	Melanoma-associated antigen 8	Tumor progression
NCCRP1	Non-specific cytotoxic cell receptor protein 1 homolog	Cell proliferation
PA2G4	Proliferation-associated protein 2G4;	Proliferation
RAB10	Ras-related protein Rab-10	Tumor progression
RAb11B	Ras-related protein Rab-11B	Tumor progression
RAB13	Ras-related protein Rab-13	Tumor progression
RAB1A	RAB1A, member RAS oncogene family	Tumor progression
RAB27A	Ras-related protein Rab-7a	Tumor progression
RAB2A	Ras-related protein Rab-2A	Tumor progression
RAB35	RAB35, member RAS oncogene family	Tumor progression
RAb6A	Ras-related protein Rab-6A	Tumor progression
RAC3	Ras-related C3 botulinum toxin substrate 3	Tumor progression
RHOC	Rho-related GTP-binding protein RhoC	Tumor progression
S100A14	Protein S100-A14	Tumor progression (through p53)
TGM3	Protein-glutamine gamma-glutamyltransferase E	tumor suppressor/inhibitor of EMT
TPT1	Tumor protein, translationally-controlled 1	Tumor progression
ZMPSTE24	CAAX prenyl protease 1 homolog	Cancer progression
ZNF383	Zinc finger protein 383	Inhibitor of MAPK pathway
RHOT2	Ras homolog family member t2	Mitochondrial GTPase
Mitochondrial integrity		
ACTA2	Actin	Mitochondria alignment
ACTB	Actin	Mitochondrial transcription
AGK	Acylglycerol kinase	Mitochondrial import (TIM22 complex)
ARPC4	Actin-related protein 2/3 complex subunit 4	Cytoarchitecture
CCHD6	MICOS complex subunit MIC25	Mitochondrial Integrity
CFL1	Cofilin-1	Cytoarchitecture
CHCHD3	MICOS complex subunit MIC19	Cristae integrity
CNP	2',3'-cyclic-nucleotide 3'-phosphodiesterase	Mitochondrial Permeability transition pore
CORO1C	Coronin-1B/1C/6	Actin binding
CSTA	Cystatin-a/b; Cystatin-A	Adhesion
DSC1	Desmocollin-1	Mitochondria alignment
DSG1	Desmoglein	Mitochondria alignment
DSP	Desmoplakin	Mitochondria alignment
FAB5	Fatty acid-binding protein	Mitochondrial integrity
FLG2	Filaggrin-2	Bundling of cytoskeletal proteins
GAPDH	Glyceraldehyde-3-phosphate dehydrogenase	Mitochondrial integrity
GGCT	Gamma-glutamylcyclotransferase	Mitochondrial Integrity
GNB1	Guanine nucleotide-binding protein G(I)/G(S)/G(T) subunit beta-1	GPCR
LMNA	Prelamin-A/C	Mitochondrial integrity through PGC1α
MTCH2	Mitochondrial carrier homolog 2	Mitochondrial integrity
PGAM5	Pgam family member 5, mitochondrial serine/threonine protein phosphatase	Mitochondrial protein quality control
PHB	Melanoma-associated antigen 8	Mitochondrial integrity
PKP1	Plakophilin-1	desmosome assembly
PRDX1	Peroxiredoxin-1	Mitochondrial oxidative stress management
PRDX2	Peroxiredoxin-2	Mitochondrial oxidative stress response
PSAP	Prosaposin	Mitochondrial proapoptotic
SAMM50	Sorting and assembly machinery component 50 homolog	criste integrity
SFXN1	Sideroflexin-1/3; Sideroflexin-1	Mitochondrial transport
SLC1A5	Neutral amino acid transporter B(0)	Amino acid transport
SLC25A1	Tricarboxylate transport protein, mitochondrial	Citrate/malate exchange mitochondrial
SLC25A10	Mitochondrial dicarboxylate carrier	Mitochondrial transport
SLC25A11	Mitochondrial 2-oxoglutarate/malate carrier protein	Mitochondrial transport
SLC25A12	Solute carrier family 25 (mitochondrial aspartate/glutamate transporter)	Mitochondrial Glutamate transporter
SLC25A13	Citrin	Mitochondrial transporter (aspartete to glutamate and proton exchange)
SLC25A22	Mitochondrial glutamate carrier 1	Mitochondrial glutamate transport
SLC25A3	Phosphate carrier protein	Mitochondrial phosphate transport
SLC25A6	Solute carrier family 25 (mitochondrial adenine nucleotide translocator)	Mitochondrial ADP/ATP translocator
SLC3A2	4F2 cell-surface antigen heavy chain	Amino acid transport
TBL2	Transducin beta-like protein 2	Mitochondria transmembrane potential
TIMM17B	Mitochondrial import inner membrane translocase subunit tim17-b	Mitochondrial Integrity
TIMM50	Mitochondrial import inner membrane translocase subunit TIM50	Mitochondrial transport
TIMMDC1	Translocase of inner mitochondrial membrane domain containing 1	Mitochondrial integrity
TMEM11	Transmembrane protein 11, mitochondrial	Mitochondrial Integrity
TMEM126A	Transmembrane protein 126A	Mitochondrial Integrity
TMEM33	Transmembrane protein 33	Mitochondrial Integrity
TOM22	Mitochondrial import receptor subunit TOM22 homolog	Mitochondrial Integrity
TOMM20	Mitochondrial import receptor subunit TOM20 homolog	Mitochondrial Integrity
TUBA1B	Tubulin alpha-1B chain	mitochondria alignment
TUBB	Tubulin beta chain	Mitochondria alignment
TUBB4B	Tubulin beta-4B chain	Mitochondria alignment
VDAC1	voltage-dependent anion-selective channel protein 1	Mitochondrial integrity
VDAC2	Voltage-dependent anion-selective channel protein 2	Mitochondrial integrity
VDAC3	Voltage-dependent anion-selective channel protein 3	Mitochondrial integrity
VIM	Vimentin	Supports mitochondrial structure and function
XRCC6	Neutral amino acid transporter B(0)	Mitochondrial inner membrane protease
YME1N1	ATP-dependent zinc metalloprotease YME1L1	Mitochondrial structural integrity via OPA1
Cell death regulation		
ANXA1	Annexin A1	Inflammation
APOD	Apolipoprotein D	Antioxidant, antiapoptotic
ATAD1	ATPase family AAA domain-containing protein 1	Links mitochondria with ER
CAS14	Caspase 12	Apoptosis
CAT	catalase	Oxidative response
CST4	Cystatin-S	Apoptosis
CTSD	Cathepsin D	Apoptosis
FLOT2	Flotillin-2	Apoptosis
GSDMA	Gasdermin-A	Pyroptosis
LTF	Lactotransferrin	Antiapoptotic
LYZ	Lysozyme C	Autophagy
NPM1	Nucleophosmin	Mitochondrla antiapoptotic
PHB2	Prohibitin 2	Mitophagy
S100A7	S100 calcium binding protein A7	Mitophagy
S100A8	Protein S100-A8	Mitophagy
S100A9	Protein S100-A9	Miophagy
VCP	Transitional endoplasmic reticulum ATPase	ER-mitochondrial Crosstalk
Chaperone and cochaperones		
HSP90AB1	Heat shock protein 90β	Chaperone
HSP90AA1	Heat shcok protein 90α	Cancer chaperone
HSPA8	Heat shock cognate 71 kDa protein	Chaperone
HSPD1	Heat shock protein family d (hsp60) member 1	Chaperone
DNAJC11	DnaJ homolog subfamily C member 11	Mitochondrial integrity
HSPA5	78 kDa glucose-regulated protein	Chaperone (ER)
HSPA9	Stress-70 protein, mitochondrial	Mitochondrial Hsp70
PPIB	Peptidyl-prolyl cis–trans isomerase B	Cyclophilin binding
Metabolism		
ACAD9	Acyl-CoA dehydrogenase family member 9,	OXPHOS (complex I)
ACSL3	Long-chain-fatty-acid–CoA ligase 3	Anabolic lipid mechanism
AGPAT5	1-acyl-sn-glycerol-3-phosphate acyltransferase epsilon	Lipid metabolism
ALDOA	Fructose-bisphosphate aldolase A	Glycolysis/gluconeogenesis
ATAD3A	ATPase family AAA domain-containing protein 3A	OXPHOS
ATP1A1	Sodium/potassium-transporting ATPase subunit alpha-1	OXPHOS
ATP5B	ATP synthase subunit β	OXPHOS
ATP5F1	ATP synthase F(0) complex subunit B1, mitochondrial	OXPHOS
ATP5O	ATP synthase subunit O, mitochondrial	OXPHOS
CALM3	Calmodulin-1	Regulation of PDH of mitochondria
COX6C	Cytochrome c oxidase subunit 6C	OXPHOS
COX7A2L	Cytochrome c oxidase subunit 7A-related protein, mitochondrial	OXPHOS
CS	Citrate synthase, mitochondrial	TCA
CYTC1	Cytochrome c1, heme protein, mitochondrial	OXPHOS
DLST	2-oxoglutarate dehydrogenase E2 component (dihydrolipoamide succinyltransferase)	TCA
GAPDH	Glyceraldehyde-3-phosphate dehydrogenase	Glycolysis
GGH	Gamma-glutamyl hydrolase	Glutamate production
MT-ATP6	ATP synthase subunit a	OXPHOS
MTCO2	Mitochondrially encoded cytochrome c oxidase ii	OXPHOS
MT-CO3	Cytochrome c oxidase subunit 3	OXPHOS
MTND5	NADH-ubiquinone oxidoreductase chain 5	OXPHOS
NDUFA10	NADH dehydrogenase [ubiquinone] 1 alpha subcomplex subunit 10	OXPHOS
NDUFA12	NADH dehydrogenase [ubiquinone] 1 alpha subcomplex subunit 12	OXPHOS
NDUFA13	NADH dehydrogenase [ubiquinone] 1 alpha subcomplex subunit 13	OXPHOS
NDUFA5	NADH dehydrogenase [ubiquinone] 1 alpha subcomplex subunit 5	OXPHOS
NDUFA6	NADH dehydrogenase [ubiquinone] 1 alpha subcomplex subunit 6	OXPHOS
NDUFA7	NADH dehydrogenase [ubiquinone] 1 alpha subcomplex subunit 7	OXPHOS
NDUFA8	NADH dehydrogenase [ubiquinone] 1 alpha subcomplex subunit 8	OXPHOS
NDUFA9	NADH dehydrogenase [ubiquinone] 1 alpha subcomplex subunit 9,	OXPHOS
NDUFB10	NADH dehydrogenase [ubiquinone] 1 beta subcomplex subunit 10;	OXPHOS
NDUFB11	NADH dehydrogenase [ubiquinone] 1 beta subcomplex subunit 11, mitochondrial	OXPHOS
NDUFB3	NADH dehydrogenase [ubiquinone] 1 beta subcomplex subunit 3	OXPHOS
NDUFB4	NADH dehydrogenase [ubiquinone] 1 beta subcomplex subunit 4	OXPHOS
NDUFB4	NADH dehydrogenase [ubiquinone] 1 beta subcomplex subunit 6	OXPHOS
NDUFB5	ADH dehydrogenase [ubiquinone] 1 beta subcomplex subunit 5, mitochondrial;	OXPHOS
NDUFB7	NADH dehydrogenase [ubiquinone] 1 beta subcomplex subunit 7	OXPHOS
NDUFB8	NADH dehydrogenase [ubiquinone] 1 beta subcomplex subunit 8, mitochondrial	OXPHOS
NDUFB9	NADH dehydrogenase [ubiquinone] 1 beta subcomplex subunit 9	OXPHOS
NDUFC2	NADH dehydrogenase [ubiquinone] 1 subunit C2	OXPHOS
NDUFS1	Mitochondrial ubiquinone oxidoreductase	OXPHOS
NDUFS2	NADH dehydrogenase [ubiquinone] iron-sulfur protein 2, mitochondrial	OXPHOS
NDUFS3	NADH dehydrogenase [ubiquinone] iron-sulfur protein 3	OXPHOS
NDUFS4	NADH dehydrogenase [ubiquinone] iron-sulfur protein 4, mitochondrial	OXPHOS
NDUFS7	NADH dehydrogenase [ubiquinone] iron-sulfur protein 7, mitochondrial	OXPHOS
NDUFS8	NADH dehydrogenase [ubiquinone] iron-sulfur protein 8, mitochondrial	OXPHOS
NDUFV1	NADH dehydrogenase [ubiquinone] flavoprotein 1	OXPHOS
PDHB	Pyruvate dehydrogenase E1 component subunit beta, mitochondrial	Mitochondrial metabolism
PHGDH	D-3-phosphoglycerate dehydrogenase	Amino acid metabolism
PKM	Pyruvate kinase m1/2	Glycolysis
SLC25A5	Solute carrier family 25 (mitochondrial adenine nucleotide translocator)	ADP/ATP exchange across the mitochondrial Membrane
SLC2A1	Solute carrier family 2, facilitated glucose transporter member 1	Glucose transporter
TMEM97	Sigma intracellular receptor 2	OXPHOS deficiency
UQCRC1	Cytochrome b-c1 complex subunit 1	OXPHOS
UQCRC2	Cytochrome b-c1 complex subunit 2, mitochondrial	OXPHOS
ZAG	Zinc-alpha-2-glycoprotein	Fatty acid oxidation
Miscellaneous functions (includes transcription)		
ATP2A2	Sarcoplasmic/endoplasmic reticulum calcium ATPase 2	ER Stress response
BCAP31	B-cell receptor-associated protein 31	ER Stress response
CALML5	Calmodulin-like protein 5	Calcium binding
CAV1	Caveolin-1	Lipid rafts
ERLIN1	Erlin-1	ER stress response and Cholesterol homeostasis
ERLIN2	Erlin-2	ER stress response and Cholesterol homeostasis
GALNT2	Polypeptide N-acetylgalactosaminyltransferase 2	Glycosylation
H2AFX	H2A histone family member X	Chromatin condensation
HIST1H3D	Histone cluster 1 H3 family member D	Chromatin/nucleosome
HIST1H3H	Histone cluster 1 H3 family member H	Chromatin/nucleosome
HIST1H3J	Histone cluster 1 H3 family member j	Chromatin/nucleosome
HIST1H4F	Histone cluster 1 H4 family member f	Nucleosome regulator
HIST1H4H	Histone cluster 1 H4 family member f	Nucleosome regulator
HIST1H4J	Histone cluster 1 H4 family member f	Nucleosome regulator
JAGN1	Protein jagunal homolog 1	ER transmembrane protein
KDELR1	ER lumen protein-retaining receptor 1	ER
MLEC	Malectin	Glycosylation
MOGS	Mannosyl-oligosaccharide glucosidase	Protein assembly (ER)
MYADM	Myeloid associated differentiation marker	Differentiation
PLOD1	Procollagen-lysine,2-oxoglutarate 5-dioxygenase 1	Collagen assembly
PLOD3	Procollagen-lysine,2-oxoglutarate 5-dioxygenase 3	Collagen processing and Cellular integrity
PSMB6	Proteasome subunit beta type-6	Proteasomal degradation
PTPLAD1	Very-long-chain (3R)-3-hydroxyacyl-CoA dehydratase 3	ER fatty acid synthesis
RPN2	Dolichyl-diphosphooligosaccharide–protein glycosyltransferase subunit 2	Glycosylation (ER)
SEC11A	SEC11 homolog A, signal peptidase complex subunit	ER
SEC22B	Vesicle-trafficking protein SEC22b	ER transport
SERPINB12	Serpin B12	Serine protease inhibitor
SERPINB3	Serpin B3	Serine protease inhibitor
SSBP1	Single-stranded DNA-binding protein, mitochondrial	Mitochondrial RNA binding
SSR4	Translocon-associated protein subunit delta	ER membrane topogenesis
STOML2	Stomatin-like protein 2	Mitochondrial biogenesis
STT3A	Dolichyl-diphosphooligosaccharide–protein glycosyltransferase subunit STT3A	Membrane function
SUB1	Activated RNA polymerase II transcriptional coactivator p15	Transcription
TADA2B	Transcriptional adapter 2-beta	Transcription
TFRC	transferrin receptor protein 1	Iron uptake
TMED2	Transmembrane emp24 domain-containing protein 2	Viral signaling
YWHAZ	14-3-3 protein zeta/delta	Signaling
RNA/DNA binding		
RPL10A	60S ribosomal protein L10a	Translation
RPL10A	Large subunit ribosomal protein l18ae	Translation
RPL11	60S ribosomal protein L11	Translation
RPL12	Large subunit ribosomal protein l12e	translation
RPL13	Large subunit ribosomal protein l13e	Translation
RPL13A	Large subunit ribosomal protein l13ae	Translation
RPL15	Large subunit ribosomal protein l15e	Translation
RPL18	60S ribosomal protein L18	Translation
RPL18A	Large subunit ribosomal protein l18ae	Translation
RPL22	Large subunit ribosomal protein l22e	Translation
RPL23	Large subunit ribosomal protein l23e	Translation
RPL27	60S ribosomal protein L27	Translation
RPL27A	Large subunit ribosomal protein l27ae	Translation
RPL28	60S ribosomal protein L28	Translation
RPL7	60S ribosomal protein L7	translation
RPL8	60S ribosomal protein L8	Translation
RPLP0	Ribosomal protein lateral stalk subunit p0	Translation
RPS14	Small subunit ribosomal protein s14e	Translation
RPS15A	Small subunit ribosomal protein s15ae	Translation
RPS16	Small subunit ribosomal protein s16e	Translation
RPS18	40S ribosomal protein S18	Translation
RPS19	40S ribosomal protein S19	Translation
RPS20	Small subunit ribosomal protein s20e	Translation
RPS25	Small subunit ribosomal protein s25e	Translation
RPL7	60S ribosomal protein L7	Ribosomal subunit
RPS3	40S ribosomal protein S3	Translation
RPS4X	40S ribosomal protein S4, X isoform	Translation
RPS5	Small subunit ribosomal protein s5e	Translation
RPS8	Small subunit ribosomal protein s8e	Translation
EEEF2	Elongation factor 2	translation elongation
EEF1A1	Elongation factor 1-alpha 1	Binds to HSF1 to regulate Hsp transcription
EEF1G	Eukaryotic translation elongation factor 1 gamma	MAVS
HNRNPM	Heterogeneous nuclear ribonucleoprotein M	Translation
PCB3	Poly(rc)-binding protein 3/4	RNA binding protein
PCBP1	Poly(rC)-binding protein 1	RNA binding protein
TUFM	Elongation factor Tu, mitochondrial	Translation
TUFM	Elongation factor Tu, mitochondrial	Translocation

These results indicated that TRAP-1 interacts with metabolic intermediates to stabilize their functions. Earlier, we showed that OE cells did not show compromised mitochondrial integrity, despite showing mitochondrial fission^30^. From these results, it is clear that TRAP-1 may stabilize mitochondrial functions. To examine this, we have examined the integrity of the intermediates of the electron transport chain (ETC) and oxidative phosphorylation (OXPHOS). The immunoblot analysis showed complex I-NADH dehydrogenase intermediates of complexes, NDUF1B; complex-II—succinate dehydrogenase, SDHB; complex-III-cytochrome C reductase, UQCRC2; complex V-ATP synthase, ATP5A are intact in OE cells (Fig. 7B–D, Fig. S3).

## Discussion

Metabolic rewiring is associated with cancer progression^46^. Hypoxia stress, chronic kidney diseases, immune cells during differentiation, diabetes, virus-induced infected host cells, etc., exhibits metabolic rewiring similar to cancer^47–50^. There are no universal patterns of metabolic rewiring,however, these pathways exhibit overlapping to a more considerable extent^51^. The well-studied metabolic alteration in cancer cells is lowered mitochondrial respiration resulting in elevated glycolysis despite oxygen availability^52^. Cellular adaptations related to oxidative imbalance, nutrition deprivation, division potential of cells, etc., will facilitate metabolic rewiring to favor cell survival in unfavorable conditions, subsequently deciding the disease progression. For this reason, understanding how different molecular networks coordinate to favor metabolic rewiring helps understand the molecular basis and develop potential inhibitors or drug candidates for treatment for various civilization-associated disorders listed above^8, 11^.

Earlier, we reported TRAP-1 OE in metabolic alterations in cancer cells. We observed TRAP-1 enhancing cellular ATP levels despite decreased mitochondrial oxygen consumption and glycolysis inhibition. The experimental evidence obtained from our previous studies indicated increased glucose, glutamine, and glutamate levels in OE cells, indicating TRAP-1 influence on both glucose and glutamine metabolisms^31^. Here, we examined the impact of TRAP-1 KD and OE on the metabolic networks of tumor cells and TRAP-1 interacting proteome. The NGS and proteomics data of KD and OE cells exposed that OE cells exhibited enhanced mitochondrial metabolism. Interestingly, glycolysis is not compromised in OE cells, indicating that TRAP-1 may favor additional metabolic pathways to increase cellular ATP levels, which is in addition to glycolysis. We also demonstrated that TRAP-1 OE promoted tumor metastasis and tumor aggression^31^, and its compromise has favored antitumor response^53^. Unlike Hsp90, TRAP-1 does not have the highly charged hinge region and the tetratricopeptide repeat motif involved in client and protein–protein interactions, respectively^54^. Therefore, TRAP-1 interacting proteins or TRAP-1-regulated metabolic networks are not clear.

Therefore, using TRAP-1 knockdown (KD) and overexpression (OE) cells, we compared their quantitative transcriptome (RNA Sequencing) and proteomic (LC–MS/MS) patterns to obtain molecular signatures that are altered in response to TRAP-1 KD or OE. The RNA sequencing and metabolome analyses of parental, KD, and OE cells highlighted increased expressions of aerobic respiration, ETC, OXPHOS, and other ATP metabolic processes, such as activation of the pentose phosphate pathway in OE cells. Although these studies provided information on potential molecular networks regulated by TRAP-1, the key players that directly associate with or depend on TRAP-1 are unclear. Subsequently, the tandem affinity system using C-TAP has provided information on potential TRAP-1 interactors regulating these processes indicating that TRAP-1 favors both mitochondria-dependent and mitochondria-independent energy metabolism. Cancer cells can survive under oxygen-limited or nutrient-starved conditions. Since they are constantly exposed to selection pressure and hypoxic stress, there should be flexibility in their metabolic pathways to meet cellular energy demands concerning changes in their microenvironment. In agreement with Hanahan and Weinberg^55^, who proposed the hallmarks of cancer, our findings integrate that TRAP-1 modulates cellular energy metabolism and probably links mitochondria-independent energy metabolism with mitochondria-dependent energy metabolism. TRAP-1 also appears to keep mitochondria functionally competent, so the metabolic needs are met without compromising the ongoing cellular activity.

Since TRAP-1 expression is associated with increased cellular complexity and conserved functions in lower eukaryotes furthered our understanding that TRAP-1 may evolutionarily have been shaped to meet energy requirements^31, 56^. Since TRAP-1 OE did not compromise mitochondrial integrity, maintaining mitochondrial integrity appears to be a prerequisite for metabolic rewiring^30^. It is understood that compromised mitochondria compromise cell fate^57^. In agreement with this, a few reports suggest that TRAP-1 favors mitochondrial metabolism^58, 59^. Therefore, our findings indicate that TRAP-1 plays a role in maintaining mitochondrial plasticity. Cellular metabolism is critical to survival, proliferation, and death since all three processes require ATP. The civilization-associated disorders such as cancer and diabetes are known to influence metabolic pathways suggesting that metabolic inhibitors may interfere with the disease progression^60^. Towards this, a few metabolic inhibitors are tested and are found to be effective. However, these metabolic inhibitors could not be used generously as anticancer agents due to a lack of tumor specificity and selectivity. Our findings suggest that TRAP-1 manipulates metabolic pathways and thus favors metabolic rewiring in cancer cells. Extending the study on understanding the role of TRAP-1 in regulating its interactors may provide additional clues on how TRAP-1 favors metabolism and tumor progression.

## Limitations of this study

- Since we have used only upregulated transcript/protein IDs for analysis, we collected the common IDs also for analysis. Further, we have used the log2 fold value of > 0.5, the transcripts/proteins that showed minimal folds were excluded from the analysis.
- The study involves the established tissue culture cells grown under controlled conditions. Extending these studies to primary tumor samples may provide additional clues/mechanisms.
- We have analyzed our IDs for only metabolic analysis and excluded the entire cellular networks. Since cellular networks function in an orchestrated manner, considering all obtained IDs may provide global networks linking TRAP-1. The study is limited to metabolism since we anticipated understanding TRAP-1’s involvement in gross cellular energy metabolism.
- However, the leads obtained in this study should be evaluated more explicitly using different cell types and cancer models to expose TRAP-1 as a potential pharmacological target to combat cancer metabolism.

## Conclusion

We demonstrated how mitochondrial chaperone TRAP-1 modulates the metabolic networks of tumor cells. The leads obtained in this study expose the potential role of TRAP-1 in regulating cellular metabolic networks resulting in disease aggression. The study also provides information on the mitochondrial and extramitochondrial roles of TRAP-1 in regulating various cellular processes. The study also emphasizes TRAP-1’s contribution to anaplerotic mechanisms supporting tumor progression. Further evaluation of the leads obtained in this study may provide mechanistic insights into TRAP-1-mediated metabolic regulation in tumor cells. Our findings suggest using TRAP-1 inhibitors alone or in combination with anticancer agents may have a favorable outcome.

## Supplementary Information

Supplementary Figure S1.

Supplementary Figure S2.

Supplementary Figure S3.

Supplementary Table S1.

Supplementary Table S2.

Supplementary Table S3.

Supplementary Table S4.

Supplementary Table S5.

## Data Availability

The data generated from this study are not publicly available. However, they will be made available upon reasonable request to assr@ccmb.res.in.
