# Supplementary figures and images for: Analysis and functional relevance of the chaperone TRAP-1 interactome in the metabolic regulation and mitochondrial integrity of cancer cells
Source: Sci Rep. 2023 May 10;13:7584. doi: 10.1038/s41598-023-34728-1 (PMC10172325; doi:10.1038/s41598-023-34728-1)

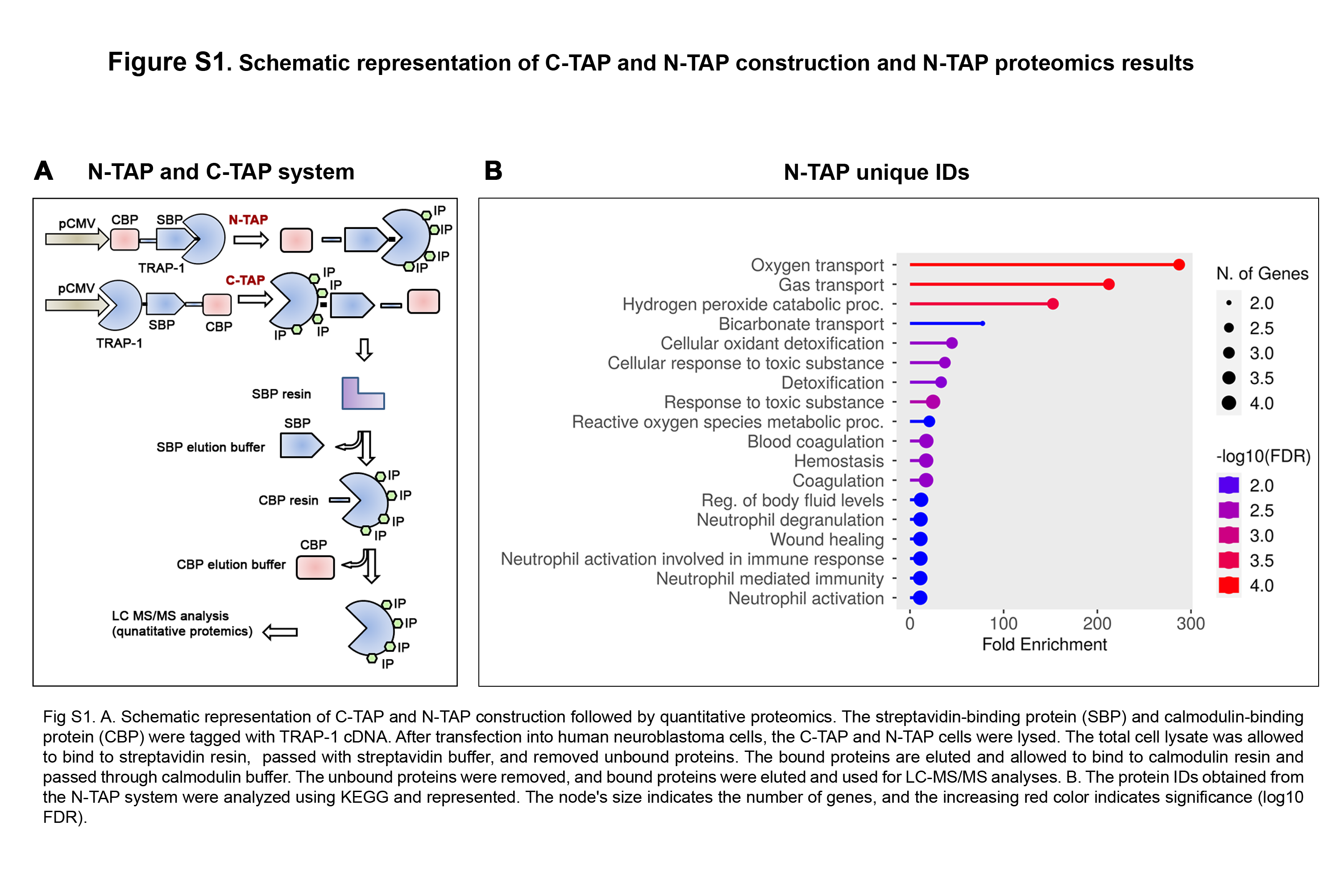

Supplement: Supplementary file 1 — Supplementary Figure S1. [file 41598_2023_34728_MOESM1_ESM.tif]

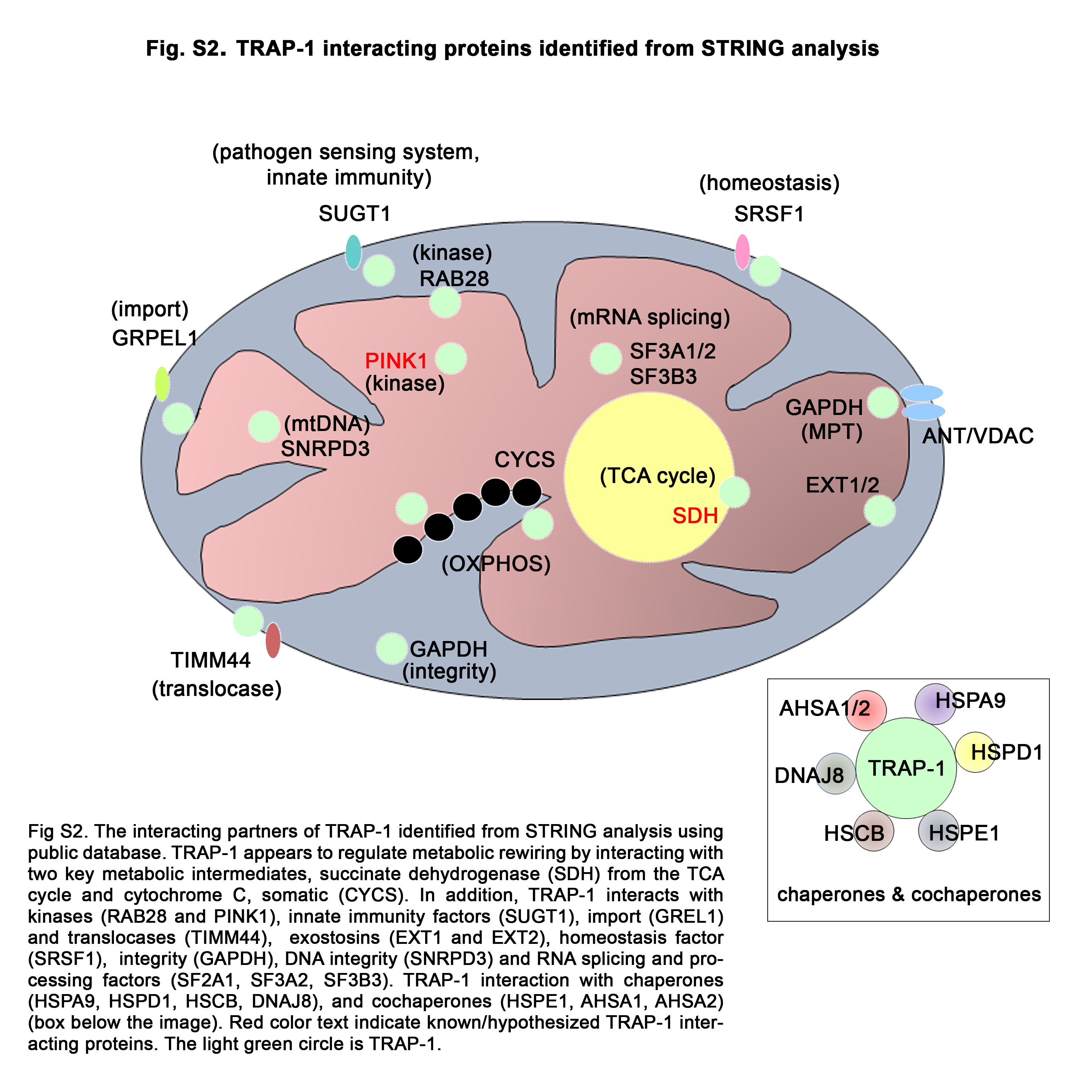

Supplement: Supplementary file 2 — Supplementary Figure S2. [file 41598_2023_34728_MOESM2_ESM.tif]

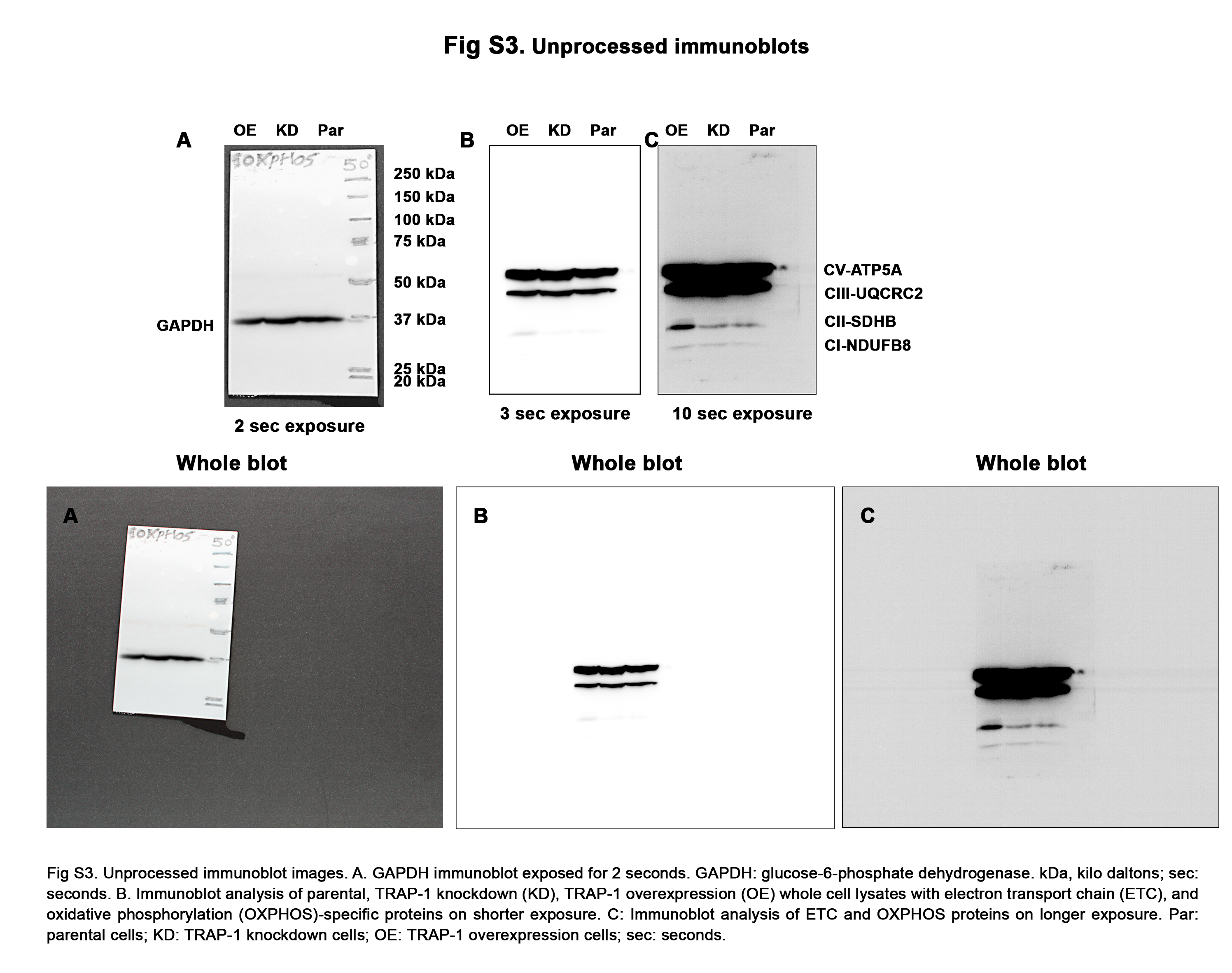

Supplement: Supplementary file 3 — Supplementary Figure S3. [file 41598_2023_34728_MOESM3_ESM.tif]
