# Supplementary material for: Analysis and functional relevance of the chaperone TRAP-1 interactome in the metabolic regulation and mitochondrial integrity of cancer cells
Source: Sci Rep. 2023 May 10;13:7584. doi: 10.1038/s41598-023-34728-1 (PMC10172325; doi:10.1038/s41598-023-34728-1)
Supplement: Supplementary file 4 — Supplementary Table S1. [file 41598_2023_34728_MOESM4_ESM.docx]

**Table S1. The PCR primers and antibodies used.**

**PCR Primers**

| **S No.** | **Gene** | **Accession** | **Primer sequence** |
| --- | --- | --- | --- |
| 1 | ATP5F1B | NM_001686.4 | Forward primer: 5'-ttgcggagactcaccccttc-3'  Reverse primer: 5'-gtccaccactgcgccaatga-3' |
| 2 | COX6C | NM_004374.4 | Forward primer: 5'-aggcgtctgcgaaatcatatgg-3'  Reverse primer: 5'-ccagccttcctcatctcctcaa-3' |
| 3 | G6PD | NM_000402.4 | Forward primer: 5'-aagcccgcctccaccaactc-3'  Reverse primer: 5'-ctgcaaaagtggcggtggtg-3' |
| 4 | IDH1 | NM_005896.4 | Forward primer: 5'-tggctctctcggcatgatga-3'  Reverse primer: 5'-aggcaaaaatggaagcaatgg-3' |
| 5 | SDHC | NM_003001.5 | Forward primer: 5'-gaaccacggccaaagaagagat-3'  Reverse primer: 5'-ccaaaaagagagacccctgcac-3' |
| 6 | PC | NM_000920.4 | Forward Primer: 5' cacggcaaagaccaccccac 3'  Reverse Primer: 5' ctcgtcgatgaactgggtgtcc 3' |
| 7 | PDHB | NM_000925.4 | Forward Primer: 5' agggtatggatgaggagctgga 3'  Reverse Primer: 5' gcacctacagcaattccagcaa 3' |

**Antibodies used**

| **S No.** | **Protein** | **Antibody dilution** | **Cat. No** |
| --- | --- | --- | --- |
| 1 | Total OXPHOS Rodent WB Antibody Cocktail | 1:5000 | ab110413 |
| 2 | GAPDH | 1:1000 | SC-47724 |
