# Supplementary material for: Analysis and functional relevance of the chaperone TRAP-1 interactome in the metabolic regulation and mitochondrial integrity of cancer cells
Source: Sci Rep. 2023 May 10;13:7584. doi: 10.1038/s41598-023-34728-1 (PMC10172325; doi:10.1038/s41598-023-34728-1)
Supplement: Supplementary file 8 — Supplementary Table S5. [file 41598_2023_34728_MOESM8_ESM.docx]

| **S NO** | **Primary interactors** | **Secondary interactors** |
| --- | --- | --- |
| 1 | AHSA1 | HSPA9, HSPD1, SUGT1 |
| 2 | AHSA2 | HSPD1, SUGT1 |
| 3 | CYCS | GAPDH, HSPA9, HSPD1, PINK1, SDHA, SDHB, SDHC, SDHD |
| 4 | DNAJC8 | HSPA9, HSPD1, SF3A1, SF3B3, SNRPD3, SRSF1 |
| 5 | EXT1 | EXT2 |
| 6 | EXT2 | EXT1 |
| 7 | GAPDH | CYCS, HSPA1, HSPD1, PINK1, SDHA, SDHB, SDHC, SRSF1 |
| 8 | HSPA9 | AHSA1, CYCS, DNAJC8, GAPDH, HSPD1, PINK1, SDHA, SDHB, SDHC, SUGT1 |
| 9 | HSPD1 | AHSA1, AHSA2, CYCS, DNAJC8, GAPDH, HSPA9, PINK1, SDHA, SDHB, SDHC, SDHD |
| 10 | PINK1 | SF3A1, SF3B3, SNRPD3, SRSF1, SUGT1, RAB28 |
| 11 | RAB28 | - |
| 12 | SDHA | CYCS, GAPDH, HSPA9, HSPD1, SDHB, SDHC, SDHD |
| 13 | SDHB | CYCS, GAPDH, HSPA9, HSPD1, SDHA, SDHC, SDHD |
| 14 | SDHC | CYCS, GAPDH, HSPA9, HSPD1, SDHA, SDHB, SDHD |
| 15 | SDHD | CYCS, SDHA, SDHB, SDHC |
| 16 | SF3A1 | DNAJC8, HSPD1, SF3B3, SNRPB3, SRSF1 |
| 17 | SF3B3 | DNAJC8, SRSF1 |
| 18 | SNRPD3 | DNAJC8, HSPD1, SF3A1, SF3B3, SRSF1 |
| 19 | SRSF1 | DNAJC8, GAPDH, HSPD1, SF3A1, SF3B1, SNRPD3 |
| 20 | SUGT1 | AHSA1, AHSA2, HSPA9, HSPD1 |

**Table S5.** **Primary and secondary interactors of TRAP-1 identified by STRING analysis.**
